# Supplementary material for: CDK6 protects epithelial ovarian cancer from platinum‐induced death via FOXO3 regulation
Source: EMBO Mol Med. 2017 Aug 4;9(10):1415–33. doi: 10.15252/emmm.201607012 (PMC5623833; doi:10.15252/emmm.201607012)
Supplement: Supplementary file 1 — Appendix [file EMMM-9-1415-s001.pdf]

APPENDIX

CDK6 protects Epithelial Ovarian Cancer from platinum-induced death *via* FOXO3 regulation

Alessandra Dall'Acqua, Maura Sonego, Ilenia Pellizzari, Ilenia Pellarin, Vincenzo Canzonieri, Sara D'Andrea, Sara Benevol, Roberto Sorio, Giorgio Giorda, Daniela Califano, Marina Bagnoli, Loredana Militello, Delia Mezzanzanica, Gennaro Chiappetta, Joshua Armenia, Barbara Belletti, Monica Schiappacassi, and Gustavo Baldassarre

Table of content:

Appendix Table S1 related to Figure 1 page 2

Appendix Table S2 related to Figure 7 page 4

Appendix Table S3 related to Material and Methods page 6

Appendix Table S4 related to Statistical Test Results page 8

Legend of Appendix Figure S1 related to Figure 1 page 13

Legend of Appendix Figure S2 related to Figure 1 page 13

Legend of Appendix Figure S3 related to Figure 6 page 14

Legend of Appendix Figure S4 related to Figure 7 page 15

Appendix Table S1: *short-hairpin RNAs*

	sh-RNA	***Name***	***TRC number***
NM_001786	CDK1 sh1	Cyclin–dependent kinase 1	TRCN0000000583
NM_001798	CDK2 sh1	Cyclin-dependent kinase 2	TRCN0000000587
	CDK2 sh2	Cyclin-dependent kinase 2	TRCN0000197236
NM_001258	CDK3 sh1	Cyclin-dependent kinase 3	TRCN0000230929
NM_000075	CDK4 sh1	Cyclin-dependent kinase 4	TRCN0000196698
	CDK4 sh2	Cyclin-dependent kinase 4	TRCN0000010520
NM_004935	CDK5 sh1	Cyclin-dependent kinase 5	TRCN0000195513
	CDK5 sh2	Cyclin-dependent kinase 5	TRCN0000194974
NM_001259	CDK6 sh1	Cyclin-dependent kinase 6	TRCN0000039747
	CDK6 sh2	Cyclin-dependent kinase 6	TRCN0000039746
	CDK6 sh3	Cyclin-dependent kinase 6	TRCN0000194893
	CDK6 sh4	Cyclin-dependent kinase 6	TRCN0000055435
NM_001799	CDK7 sh1	Cyclin-dependent kinase 7	TRCN0000000592
	CDK7 sh2	Cyclin-dependent kinase 7	TRCN0000196306
NM_001260	CDK8 sh1	Cyclin-dependent kinase 8	TRCN0000196702
	CDK8 sh2	Cyclin-dependent kinase 8	TRCN0000199980
NM_001261	CDK9 sh1	Cyclin-dependent kinase 9	TRCN0000000495
	CDK9 sh2	Cyclin-dependent kinase 9	TRCN0000000497
NM_003674	CDK10 sh1	Cyclin-dependent kinase 10	TRCN0000199232
NM_052987	CDK10 sh2	Cyclin-dependent kinase 10	TRCN0000001822
NM_024011	CDK11A sh1	Cyclin-dependent kinase 11A	TRCN0000006991
	CDK11A sh2	Cyclin-dependent kinase 11A	TRCN0000006992
NM_016507	CDK12 sh1	Cyclin-dependent kinase 12	TRCN0000001795
	CDK12 sh2	Cyclin-dependent kinase 12	TRCN0000001798
NM_003718	CDK13 sh1	Cyclin-dependent kinase 13	TRCN0000000704
NM_012395	CDK14 sh1	Cyclin-dependent kinase 14	TRCN0000002366
	CDK14 sh2	Cyclin-dependent kinase 14	TRCN0000002367
NM_139158	CDK15 sh1	Cyclin-dependent kinase 15	TRCN0000002096
	CDK15 sh2	Cyclin-dependent kinase 15	TRCN0000002097
NM_033018	CDK16 sh1	Cyclin-dependent kinase 16	TRCN0000197222
	CDK16 sh2	Cyclin-dependent kinase 16	TRCN0000010251
NM_002595	CDK17 sh1	Cyclin-dependent kinase 17	TRCN0000196448
	CDK17 sh2	Cyclin-dependent kinase 17	TRCN0000194654
NM_002596	CDK18 sh1	Cyclin-dependent kinase 18	TRCN0000006333
	CDK18 sh2	Cyclin-dependent kinase 18	TRCN0000006335
NM_015076	CDK19 sh1	Cyclin-dependent kinase 19	TRCN0000003140
NM_ 178432	CDK20 sh1	Cyclin-dependent kinase 20	TRCN0000199977
	CDK20 sh2	Cyclin-dependent kinase 20	TRCN0000199297
NM_004196	CDKL1 sh1	Cyclin-dependent kinase- like 1	TRCN0000006072
	CDKL1 sh2	Cyclin-dependent kinase- like 1	TRCN0000006073
NM_016508	CDKL3 sh1	Cyclin-dependent kinase-like 3	TRCN0000002376
	CDKL3 sh2	Cyclin-dependent kinase-like 3	TRCN0000002377
XM_293029	CDKL4 sh1	Cyclin-dependent kinase-like 4	TRCN0000021520
	CDKL4 sh2	Cyclin-dependent kinase-like 4	TRCN0000021521

NM_007056	SFRS16 sh1	serine/arginine-rich splicing factor 16	TRCN0000286219
	SFRS16 sh2	serine/arginine-rich splicing factor 16	TRCN0000286220
NM_198102	TRA2A sh1	transformer 2 alpha homolog	TRCN0000293989
	TRA2A sh2	transformer 2 alpha homolog	TRCN0000286623
NM_004593	TRA2B sh1	transformer 2 beta homolog	TRCN0000314984
	TRA2B sh2	transformer 2 beta homolog	TRCN0000314919
NM_011358	SFRS2 sh1	serine/arginine-rich splicing factor 2	TRCN0000000109
	SFRS2 sh2	serine/arginine-rich splicing factor 2	TRCN0000000084
NM_133477	SYNP2 sh1	synaptopodin 2	TRCN0000140276
	SYNP2 sh2	synaptopodin 2	TRCN0000138984
NM_080743	SFRS12 sh1	serine/arginine-rich splicing factor 12	TRCN0000151704
	SFRS12 sh2	serine/arginine-rich splicing factor 12	TRCN0000151194
NM_030751	ZEB1 sh1	zinc finger E-box binding homeobox 1	TRCN0000364631
	ZEB1 sh2	zinc finger E-box binding homeobox 1	TRCN0000017565
NM_001455	FOXO3a sh1	Forkhead O transcription factor 3a	TRCN0000010334
	FOXO3a sh2	Forkhead O transcription factor 3a	TRCN0000040098
	FOXO3a sh3	Forkhead O transcription factor 3a	TRCN0000040099
	FOXO3a sh4	Forkhead O transcription factor 3a	TRCN0000235488
	FOXO3a sh5	Forkhead O transcription factor 3a	TRCN0000235489
NM_007631	Cyc D1 sh1	Cyclin D1	TRCN0000295873
NM_001760	Cyc D3 sh1	Cyclin D3	TRCN0000003828
NM_001184	ATR sh1	Ataxia-telangiectasia Rad3-related	TRCN0000196538
	sh-ctrl	control	SHC002

Appendix Table S1: Table S1 reports the shRNAs used in this work. Accession report the GeneBank accession number of each silenced gene. shRNA indicate the symbol of the knocked genes and the shRNA number. TRC indicates the RNA consortium target sequence number.

Appendix Table S2: *Data of ovarian cancer patients*

Number	***Tumor***	***Histotype***	***Grade***	***CT/Neoadjuvant***	***TNM***
4	Primary	Mucinous	Borderline		pT3N1c
6	Primary	Serous	G3		pT2N1M1
7a	Primary	Serous	G3		pT3cN1
7b	Primary	Serous	G3		pT3cN1
8a	Primary	Serous	G3		pT3N0M1
8a1	Primary	Serous	G3		pT3N0M1
11	Primary	Serous	G3		pT3cNx
14	Primary	Serous	Borderline		pT3aNo
15	Primary	Serous	G2		pT3cN1M1
18	Primary	Serous	G1		pT2b
21	Primary	Serous	G3		pT3c
22a	Primary	Serous	G3		pT3Nx
22b	Primary	Serous	G3		pT3Nx
23a	Primary	Serous	G3		pT3cN1
23b	Primary	Serous	G3		pT3cN1
24	Primary	Serous	G3		pT3cNx
27a	Primary	Serous	G3		pT3cN1
28a	Primary	Serous	G3		pT3cN1M1
29	Primary	Serous	G3		pT3cNx
30	Primary	Serous	G3		pT3cNo
31	Primary	Serous	G3		pT3cN1
34	Primary	Serous	G3		pT3b
35a	Primary	Serous	G3		pT3cN1
37	Primary	Serous	G3		pT3c
38a	Primary	Serous	G3		pT2cN1M1
38b	Primary	Serous	G3		pT2cN1M1
40	Primary	Serous	G2		pT3cN0M1
41	Primary	Serous	G3		pT3cN1
42	Primary	Serous	G3		pT3c
43	Primary	Serous	G3		pT3b
51	Primary	Serous	G2/3		pT3c
51asc	Primary	Serous	G2/3		pT3c
57a	Primary	Serous	G2		pT3c
57b	Primary	Serous	G2		pT3c
56	Primary	Serous	G3		pT2c
60	Primary	Serous	G3		pT3cN1
63	Primary	Serous	G3		pT1cNx
64	Primary	Serous	G3		pT3cN1
71a	Primary	Serous	G3		pT3cN1
71b	Primary	Serous	G3		pT3cN1
73	Primary	Serous	G3		pT1cN1
76	Primary	Serous	G3		pT3c
79	Primary	Serous	G2		pT2c
80	Primary	Serous	G3		pT3cN1M1
83	Primary	Serous	G3		pT3cN1

84	Primary	Serous	G3		pT3bN0
**85**	**Primary**	**Undifferentiated**	**G3**		**pT3cN1**
86a1	Primary	Serous	G3		pT3c
86a4	Primary	Serous	G3		pT3c
86b	Primary	Serous	G3		pT3c
88a	Primary	Serous	G3		pT3cM1
88c	Primary	Serous	G3		pT3cM1
9	Primary	Serous	n.a.	Yes	ypT2aN0
26	Primary	Serous	n.a.	Yes	ypT3cN1
44a1	Primary	Serous	G3	Yes	ypT3cNx
44a2	Primary	Serous	G3	Yes	ypT3cNx
52	Primary	Serous	G3	Yes	ypT2bN1
59	Primary	Serous	G3	Yes	ypT3c
**77**	**Primary**	**Serous**	**n.a.**	**Yes**	**pT3c**
81	Primary	Serous	n.a.	Yes	pT3cN1M1
**91a**	**Primary**	**Serous**	**n.a.**	**Yes**	**ypT3c**
**66**	**Recurrence Ascites**	**Serous**	**n.a**		
67	Recurrence	Serous	n.a.		
68	Recurrence	Serous	n.a.		
69	Recurrence	Serous	n.a.		
70	Recurrence	Serous	n.a.		
**72**	**Recurrence**	**Serous**	**n.a.**		

Appendix Table S2: Histo-pathological and clinical data of ovarian cancer patients. Appendix Table S2 reports grade, histotype and chemotherapy (when applied) of primary and recurrent tumor samples analyzed in Figure 7 and Appendix Figure 9 (n.a.: not available). In bold, the samples from which primary EOC cultures were established and tested for their survival after platinum and platinum + PD treatments are highlighted (Fig 7C and Appendix Fig S7C).

Appendix Table 3: *Primers*

Primer	*Sequence 5' – 3'*
CDK6 R31C *forward*	GAAGGTGTTCAAGGCCTGCGACTTGAAGAACGGAGG
CDK6 R31C *reverse*	CCTCCGTTCTTCAAGTCGCAGGCCTTGAACACCTTC
CDK6 D163N *forward*	GACAAATAAAACTCGCTAACTTCGGCCTTGCCCGCATC
CDK6 D163N *reverse*	GATGCGGGCAAGGCCGAAGTTAGCGAGTTTTATTTGTC
FOXO3 S294A *forward*	CTCCAAGTGGCCTGGCGCCCCCACGTCACGCAGCAGTG
FOXO3 S294A *reverse*	CTCACTGCTGCGTGACGTGGGGGCGCCAGGCCACTTGGAG
FOXO3 S344A *forward*	GATGATGCGCCTCTCGCGCCCATGCTCTACAGCAGC
FOXO3 S344A *reverse*	GCTGCTGTAGAGCATGGGCGCGAGAGGCGCATCATC
FOXO3 S325A *forward*	GTGGCCGCCTGGCGCCCATCATGGC
FOXO3 S325A *reverse*	GCCATGATGGGCGCCAGGCGGCCAC
FOXO3 S325E *forward*	GTGGCCGCCTGGAGCCCATCATGGC
FOXO3 S325E *reverse*	GCCATGATGGGCTCCAGGCGGCCAC
FOXO3 NT *forward*	GGATCCATGGCAGAGGCACCGGCT
FOXO3 CT *reverse*	GCGGCCGCTCAGCCTGGCACCCAG
FOXO3 CT1 *forward*	GGATCCCGGAACGTGATGCTTCGCAAT
FOXO3 CT3 *forward*	GGATCCACCAGCTCCTTTAACAGCACGGT
FOXO3 CT4 *forward*	GGATCCCTCTACAGCAGCTCAGCCAGC
FOXO3 CT5 *forward*	GGATCCGCCAGCACAGTCAGTGGCCGC
FOXO3 CT6 *forward*	GGATCCGCTGACGACAGTCCCTCCCAG
FOXO3 CT7 *forward*	GGATCCGTGGAACTGCCACGGCTGACT
FOXO3 (345-674) delta *forward*	AAGCTTCCCATGCTCTACAGCAGC
FOXO3 (1-314) delta *reverse*	AAGCTTATTGGTGCGTGAACGGAA
ATR promoter -1200/-1000 *forward*	AACATAGCAAGACCTTGTCTCTA
ATR promoter -1200/-1000 *reverse*	ACTCTGTTGTTCAGGTTCTAGAG
ATR promoter -1000/-800 *forward*	AAGACAATGTATCTAACAAAAAAA
ATR promoter -1000/-800 *reverse*	GAAAAAAGTGATGGAATGACAGCC
ATR promoter -600/-400 *forward*	TGCGGATGCCCGTAATGGTG
ATR promoter -600/-400 *reverse*	TCTGAGAGATACAGGCCAAAAG
ATR promoter -400/-200 *forward*	AAGAGGGACAAGAGCGGTGG
ATR promoter -400/-200 *reverse*	AAGCCTGGGAGGCACAGAGA
ATR promoter -200/0 *forward*	GAGAACAGCAGAGTCTGGCC
ATR promoter -200/0 *reverse*	GCCGCTACTGGCCCCGCTT
ATR promoter -1000/-805 *reverse*	AAGTGATGGAATGACAGCCAAATA
ATR promoter -890/-710 *forward*	ATGACAGAGTGGTGTTCAAGGC
ATR promoter -890/-710 *reverse*	AAGTTATGTAGTTAATCACTCAAAGTC
ATR promoter -739/-510 *forward*	CATGACTTTGAGTGATTAACTACA
ATR promoter -739/-510 *reverse*	AATATTTGAAAATGGGGTTGGAAT
ATR *forward*	CGCTGAACTGTACGTGGAAA
ATR *reverse*	CAATTAGTGCCTGGTGAACATC
Pol2a *forward*	GCAAATTCACCAAGAGAGACG
Pol2a *reverse*	CACGTCGACAGGAACATCAG
CDK1 *forward*	TGGATCTGAAGAAATACTTGGATTCTA
CDK1 *reverse*	CAATCCCCTGTAGGATTTGG
CDK2 *forward*	CCTCCTGGGCTGCAAATA
CDK2 *reverse*	CAGAATCTCCAGGGAATAGGG

CDK3 *forward*	TGGTGACACTGTGGTATCGC
CDK3 *reverse*	TCACCAGGAAACAGGGCTT
CDK4 *forward*	GTGCAGTCGGTGGTACCTG
CDK4 *reverse*	TTCGCTTGTGTGGGTTAAAA
CDK6 *forward*	TGATCAACTAGGAAAAATCTTGGAC
CDK6 *reverse*	GGCAACATCTCTAGGCCAGT
CDK7 *forward*	CCATGTGCTCGAATTACGG
CDK7 *reverse*	CTTGGCAGCTGACATCCAG
CDK8 *forward*	AAGCTGCTTACCATGGACCC
CDK8 *reverse*	TGACAACCGGCAAAAACGTC
CDK9 *forward*	TTCGGGGAGGTGTTCAAG
CDK9 *reverse*	ATCTCCCGCAAGGCTGTAAT
CDK10 *forward*	TCATGTACGACCCTAAGAAAAGG
CDK10 *reverse*	CACCACTGATCGGAAGACG
CDK11A *forward*	AAGTGTTCAAGGAGCTGGGG
CDK11A *reverse*	TGTTCATGAGGTCGAAGCCC
CDK12 *forward*	GAACTCCCACAATGCCACA
CDK12 *reverse*	CTCTGGTGGAAGAATGTGAGG
CDK13 *forward*	ATCTCAGGGCAGCTCAAATG
CDK13 *reverse*	TTGCCAATTCACTGTGGTTTA
CDK14 *forward*	CAGTATACAAAGGGAAAAGCAAGG
CDK14 *reverse*	CCCTGATAGCTGTGAAAGGTG
CDK15 *forward*	TTTGCTGGGAGCCACTGAAT
CDK15 *reverse*	CCCAGCACCTCCCAGATTTT
CDK16 *forward*	GCAGTGACCCTGGAGAGG
CDK16 *reverse*	TCAAGTCCTCGTGCACAATC
CDK17 *forward*	TGTTATTGGAGGGAGCCTTG
CDK17 *reverse*	TCTGATCCCATTTTTAGATTTTCA
CDK18 *forward*	GCCGGGTATAAGGAGCAAA
CDK18 *reverse*	GGAGAAACGGCGCTTAAAGT
CDK19 *forward*	CTTTCTCACAGTGACAGGAAGGT
CDK19 *reverse*	TTTGATGCACGGTGAAACTT
CDK20 *forward*	TCCAAGGCTCTCCTCCATCA
CDK20 *reverse*	TCTGGGTTCAACAGCGACTC
CDKL1 *forward*	CTGTCAGGAGTGCCTCTGTG
CDKL1 *reverse*	GCTAAACACTTGCTGGTGCC
CDKL3 *forward*	AGAAAGAGCCGAAAGCACCA
CDKL3 *reverse*	AGGATGCTGCTGCCAGTTAG
CDKL4 *forward*	ACTGTGGCCTGGAAAATCAGA
CDKL4 *reverse*	TGATGCCATGGAAAAACCCG

Appendix Table S3: Nucleotide sequence of the primers used for site-directed mutagenesis of FOXO3a and CDK6 cDNAs, those used to generate FOXO3a deletion mutants, those used for ATR promoter fragments' amplification and those used to evaluate the normalized expression of CDKs and ATR mRNA.

Appendix Table 4: *Exact P value*

Figure 1	
**Figure 1B**	**p value**
sh-ctrl vs sh1	0,0016
sh-ctrl vs sh2	0,0011
sh-ctrl vs sh3	0,0015
sh-ctrl vehicle vs sh1 CBDCA	0,0020
**Figure 1D**	**p value**
sh-ctrl vehicle vs sh-CDK6 CDDP	0,0026
sh-ctrl CDDP vs sh-CDK6 CDDP	0,0009
sh-CDK6 vehicle vs sh-CDK6 CDDP	0,0011
**Figure 1H**	**p value**
sh-ctrl vs sh-CDK6 72h	< 0,0001
sh-ctrl vs sh-CDK6 96h	< 0,0001
sh-ctrl vs sh-CDK6 vehicle	0,0010
sh-ctrl vs sh-CDK6 CBDCA	0,0056
**Figure 2**	
**Figure 2A**	**p value**
e.v. vs R31C	0,0094
WT vs R31C	0,0019
WT vs D163N	0,0347
R31C vs D163N	0,0007
**Figure 2E**	**p value**
0% vs 10%	0,0426
0% vs 20%	0,0013
**Figure 3**	
**Figure 3B**	**p value**
vehicle vs CBDCA+PD	0,0008
CBDCA vs CBDCA+PD	0,0004
PD vs CBDCA+PD	0,0320
**Figure 3C**	**p value**
vehicle vs CBDCA+PD	0,0050
CBDCA vs CBDCA+PD	0,0030
PD vs CBDCA+PD	0,0178
**Figure 3F**	**p value**
vehicle vs CBDCA+PD	< 0,0001
CBDCA vs CBDCA+PD	< 0,0001
PD vs CBDCA+PD	< 0,0001
**Figure 3H**	**p value**
sh-ctrl vs sh-CDK6	0,0209

Figure 4	
**Figure 4A**	**P value**
sh-ctrl vs sh2	0,0030
sh-ctrl vs sh4	0,0384
**Figure 4C**	**P value**
sh-ctrl vs sh-CDK6 e.v. (0.1)	0,0029
sh-ctrl vs sh-CDK6 e.v. (0.3)	0,0025
sh-ctrl vs sh-CDK6 e.v. (1.5)	0,0001
sh-ctrl vs sh-CDK6 e.v. (3.0)	0,0141
sh-ctrl vs sh-CDK6 e.v. (15.0)	0,0149
sh-ctrl vs sh-CDK6 e.v. (30.0)	0,0010
sh-ctrl vs sh-CDK6 e.v. (150.0)	0,0011
sh-CDK6 e.v vs sh-CDK6 FOXO3 WT (0.1)	0,0086
sh-CDK6 e.v vs sh-CDK6 FOXO3 WT (0.3)	0,0028
sh-CDK6 e.v vs sh-CDK6 FOXO3 WT (1.5)	0,0027
sh-CDK6 e.v vs sh-CDK6 FOXO3 WT (3.0)	0,0277
sh-CDK6 e.v vs sh-CDK6 FOXO3 WT (15.0)	0,0072
sh-CDK6 e.v vs sh-CDK6 FOXO3 WT (30.0)	0,0073
sh-CDK6 e.v vs sh-CDK6 FOXO3 WT e.v. (150.0)	0,0227
**Figure 5**	
**Figure 5A**	**P value**
sh-ctrl vs sh-CDK6 (2h)	0,0010
sh-ctrl vs sh-CDK6 (4h)	0,0005
**Figure 5B**	**P value**
WT vs S325A (2h)	0,0047
WT VS S325A (4h)	0,0001
**Figure 5E**	**P value**
WT vs S352A (P)	0,0005
WT vs S352A (R)	0,0002
**Figure 5F**	**P value**
WT vs S352A (P)	0,0066
**Figure 5G**	**P value**
sh-ctrl vs sh-CDK6+e.v. (1.5)	0,0314
sh-CDK6+e.v. vs sh-CDK6+WT (1.5)	< 0,0001
sh-CDK6+WT vs sh-CDK6+352A (1.5)	0,0404
sh-CDK6+e.v. vs sh-CDK6+WT (15.0)	0,0333
sh-CDK6+WT vs sh-CDK6+352A (15.0)	0,0066
**Figure 5I**	**P value**
vehicle vs CBDCA+PD	< 0,0001
CBDCA vs CBDCA+PD	< 0,0001
PD vs CBDCA+PD	< 0,0001

Figure 6	
**Figure 6A**	**P value**
sh-ctrl vs sh-FOXO3 (ATR 3h)	0,0470
sh-ctrl vs sh-CDK6 (ATR 3h)	0,0043
sh-ctrl vs sh-FOXO3 (ATR 16h)	0,0023
sh-ctrl vs sh-CDK6 (ATR 16h)	0,0111
sh-ctrl vs sh-FOXO3 (FOXO3 3h)	0,0096
sh-ctrl vs sh-FOXO3 (FOXO3 16h)	0,0022
sh-ctrl vs sh-CDK6 (FOXO3 16h)	0,0285

Appendix Figure S1	
**S1B**	**P value**
sh-ctrl vs sh-CDK6	< 0,0001
sh-ctrl vs sh-CDK17	0,0137
**S1C**	**p value**
sh-ctrl vs sh1-CDK1	0,0038
sh-ctrl vs sh1-CDK2	0,0228
sh-ctrl vs sh2-CDK2	0,0089
sh-ctrl vs sh1-CDK3	0,0206
sh-ctrl vs sh1-CDK4	0,0037
sh-ctrl vs sh1-CDK5	0,0029
sh-ctrl vs sh2-CDK5	0,0104
sh-ctrl vs sh1-CDK6	0,0004
sh-ctrl vs sh1-CDK7	0,0201
sh-ctrl vs sh2-CDK7	0,0166
sh-ctrl vs sh2-CDK8	0,0490
sh-ctrl vs sh1-CDK9	0,0363
sh-ctrl vs sh2-CDK9	0,0289
sh-ctrl vs sh1-CDK10	0,0217
sh-ctrl vs sh2-CDK10	0,0342
sh-ctrl vs sh2-CDK11A	0,0311
sh-ctrl vs sh1-CDK14	0,0372
sh-ctrl vs sh1-CDK17	0,0318
sh-ctrl vs sh1-CDK20	0,0352
sh-ctrl vs sh2-CDK20	0,0352
**S1D**	**P value**
cells vs sh1-CDK6	0,0042
sh-ctrl vs sh1-CDK6	0,0001
**Appendix Figure S2**	
**S2B**	**P value**
sh-ctrl vehicle vs sh-CDK6 CDDP	0,0285
sh-CDK6 vehicle vs sh-CDK6 CDDP	0,0337

Appendix Figure S3	
**S3E**	**P value**
sh-ctrl vs sh-CDK6 (2h)	0,0439
sh-ctrl vs sh-FOXO3 (2h)	0,0164
**Appendix Figure S4**	
**S4A**	**P value**
D1 primary vs D3 pretreated	< 0.0001
D3 primary vs D3 pretreated	0,0019
D1 pretreated vs D3 pretreated	< 0.0001
**S4B**	**P value**
correlation FOXO3 vs CDK6	0,0003
correlation FOXO3 vs ATR	0,0485
correlation ATR vs CDK6	0,0461
correlation ATR vs Cyclin D3	0,0002

FIGURE EV1	
**EV1E**	**P value**
empty vector vs CDK6 WT (9.0)	0,0154
empty vector vs CDK6 WT (30.0)	0,0008
**FIGURE EV2**	
**EV2B**	**P value**
vehicle vs CBDCA+PD	0,0134
**EV2C**	**P value**
vehicle vs CBDCA	0,0102
vehicle vs PD	0,0003
vehicle vs CBDCA+PD	< 0,0001
CBDCA vs CBDCA+PD	0,0007
PD vs CBDCA+PD	0,0032
**EV2E**	**P value**
CBDCA vs CBDCA+PD	< 0,0001
PD vs CBDCA+PD	0,0002
**EV2F**	**P value**
CBDCA vs CBDCA+PD	0,0002
PD vs CBDCA+PD	< 0,0001
**EV2G**	**P value**
CBDCA vs CBDCA+PD	0,0456
**EV2I**	**P value**
vehicle vs CBDCA+PD	0,0006
**EV2J**	**P value**
vehicle vs CBDCA+PD	0,0487

FIGURE EV3	
**EV3B**	**P value**
e.v vs D263N	0,0458
**EV3G**	**P value**
parental vs pt_resistant 2	0,0199
**EV3I**	**P value**
vehicle vs PD (vehicle)	0,0055
vehicle vs PD (CBDCA)	0,0003
PD (vehicle) vs PD (CBDCA)	0,0359
**FIGURE EV4**	
**EV4C**	**P value**
sh-ctrl vs sh-FOXO3	0,0100
sh-ctrl vs sh-CDK6	0,0050

Appendix Table S4: The table reports the exact P values calculated for each test in the corresponding figure panels.

Appendix Figure S1. CDK6 knock-down sensitizes MDAH cells to platinum induced cell death.

A Dose response curve on MDAH cells transduced with Control (ctrl) shRNA and treated with increasing doses of CBDCA for 16 hours used to calculate the CDBCA IC50 in control cells (3 biological replicates).

B Screening results: data represent the mean ± SD of 5 independent experiments performed in triplicate and are expressed as survival ratio between of CBDCA (140 μg/ml for 16 hours) treated and untreated cells. Red line indicates the pre specified cut off of significance that corresponds to the double of the standard deviation observed in cells transduced with ctrl shRNA (two-sided, unpaired *t* test).

C Quantitative RT-PCR (qRT-PCR) analyses of the expression of CDKs expressed in MDAH cells at a detectable level by qRT-PCR (i.e. ≥ 0.00015 amol/ng of total RNA that correspond to a normalized expression ≥ 0.1) in cells transduced with the indicated shRNAs and evaluated 48 hours post transduction. Experiements were performed in triplicate. Data are expressed as normalized levels to housekeeping pol2A (two-sided, unpaired *t* test).

D Viability of MDAH cells transduced with the indicated shRNAs and treated with CBDCA (140 μg/ml) for 16 hours. WB shows CDK6 and CDK4 expression in the corresponding cell lysates. Data represent the mean ± SD of 3 independent experiments performed in duplicate (two-sided, unpaired *t* test). Vinculin was used as loading control.

Exact P values of Appendix Fig S1B-D are reported in Appendix Table S4.

Appendix Figure S2. CDK6 knock-down sensitizes SKOV3ip cells to platinum induced cell death.

A Table summarizing CDDP or CBDCA IC50 of SKOV3ip cells transduced with Control (ctrl) or CDK6-specific shRNAs. Results are expressed as percentage of viable cells respect to untreated cells and the resulted IC50 (half maximal inhibitory concentrations) are reported (n = 3, performed in triplicate).

B Colony assay and its quantification performed on SKOV3ip cells transduced as indicated and treated or not with CDDP for 72 hours and then released for 72 hours. Data represent the mean ± SD of 2 independent experiments performed in triplicate (two-sided, unpaired *t* test).

C Growth curve of control or CDK6 silenced SKOV3ip cells. The corresponding cell lysates were analyzed by western blot. Vinculin was used as loading control. Data represent the mean ± SD of two independent experiment performed in triplicate.

D SA-β-Galactosidase positive cells/field in SKOV3ip cells transduced as indicated and stained 72 and 96 hours post transduction.

Exact P values of Appendix Fig S2B is reported in Appendix Table S4.

Appendix Figure S3. FOXO3 controls ATR transcription and prevents premature chromosome condensation.

A Schematic representation of ATR promoter. The 3 putative FOXO3 binding sites (colored bars) located at the position indicated in the colored boxes are shown. Amplified fragments of the different regions of FOXO3 promoter are reported.

B,C Experimental design of ChIP experiments shown in Fig 6E and F and Appendix Fig S3F, evaluating the association of overexpressed (B) or endogenous (C) FOXO3 or CDK6 proteins with the ATR promoter.

D Typical amplification reaction of genomic DNA (lower panel) or DNA recover from ChIP using an anti-FOXO3 antibody using chromatin from MDAH cells treated with CDDP for 3 hours (R 0h, upper panel) and treated with CDDP for 3 hours and then released for 3 additional hours (R 3h, middle panel). Only the region encompassing nucleotide -1000/-800 (predicted site 1) are amplified in DNA recover from ChIP using the anti-FOXO3 antibody.

E Expression of ATR mRNA in MDAH cells transduced with the indicated shRNAs and then treated with 15 μg/ml CDDP for 2 hours (2h) and then allowed to repair for 3 hours (R 3h). Data are expressed as fold respect to the same cells treated with vehicle at the same time point.

F Enrichment of ATR promoter in CDK6-IPs in Chromatin immunoprecipitation (ChIP) analysis using cells treated with vehicle (V) or with CDDP (P) and then released (R) for 3 hours. Data represent the mean of 3 biological replicates and are expressed as fold enrichment respect to control IgG (two-sided, unpaired *t* test).

G Representative images of metaphases (mitotic spreads) of MDAH cells transduced with control (ctrl) or CDK6-specific shRNAs. Graph shows % of PCC respect to total nuclei in the same

experiment. On the right, is reported the expression of CDK6 analyzed by western blot. Vinculin was used as loading control.

Exact P values of Appendix Fig S3E is reported in Appendix Table S4.

Appendix Figure S4. High expression of CDK6 and FOXO3a predicts low survival in EOC patients.

A Quantification of Cyclin D1/D3 expression in tumor samples described in Appendix Table S2. Results are presented as scattered dot plot and represent the normalized expression respect to vinculin. Bars indicates mean 95% CI. Significance was calculated using two-sided, unpaired *t* test.

B Graphs show the Pearson's analyses evaluating the correlation between FOXO3 and CDK6 or ATR expression or between ATR and CDK6 or CyclinD3 expression in samples from platinum treated patients (i.e. patients with recurrent disease or treated with neo-adjuvant chemotherapy).

C CDDP IC50 of primary cultures derived from the indicated tumors in the presence or not of PD (3 biological replicates).

D Table summarizing univariate and multivariate analysis (Cox regression) of PFS for clinical and biological variables in high grade cases (n = 210, number of events = 151) of Tothill dataset.

E Kaplan-Meier survival curves evaluating the prognostic significance of FOXO3, ATR and CDK6-Cyclin D3 or CDK6-Cyclin D1 combination in predicting the PFS of EOC patients with the online tools Kaplan-Meier Plotter (KMP: n = 1307; events = 484; log-rank test).

In D and E HR = Hazard Ratio; CI = Confident Interval.

Exact P values of Appendix Fig S4A and B are reported in Appendix Table S4.

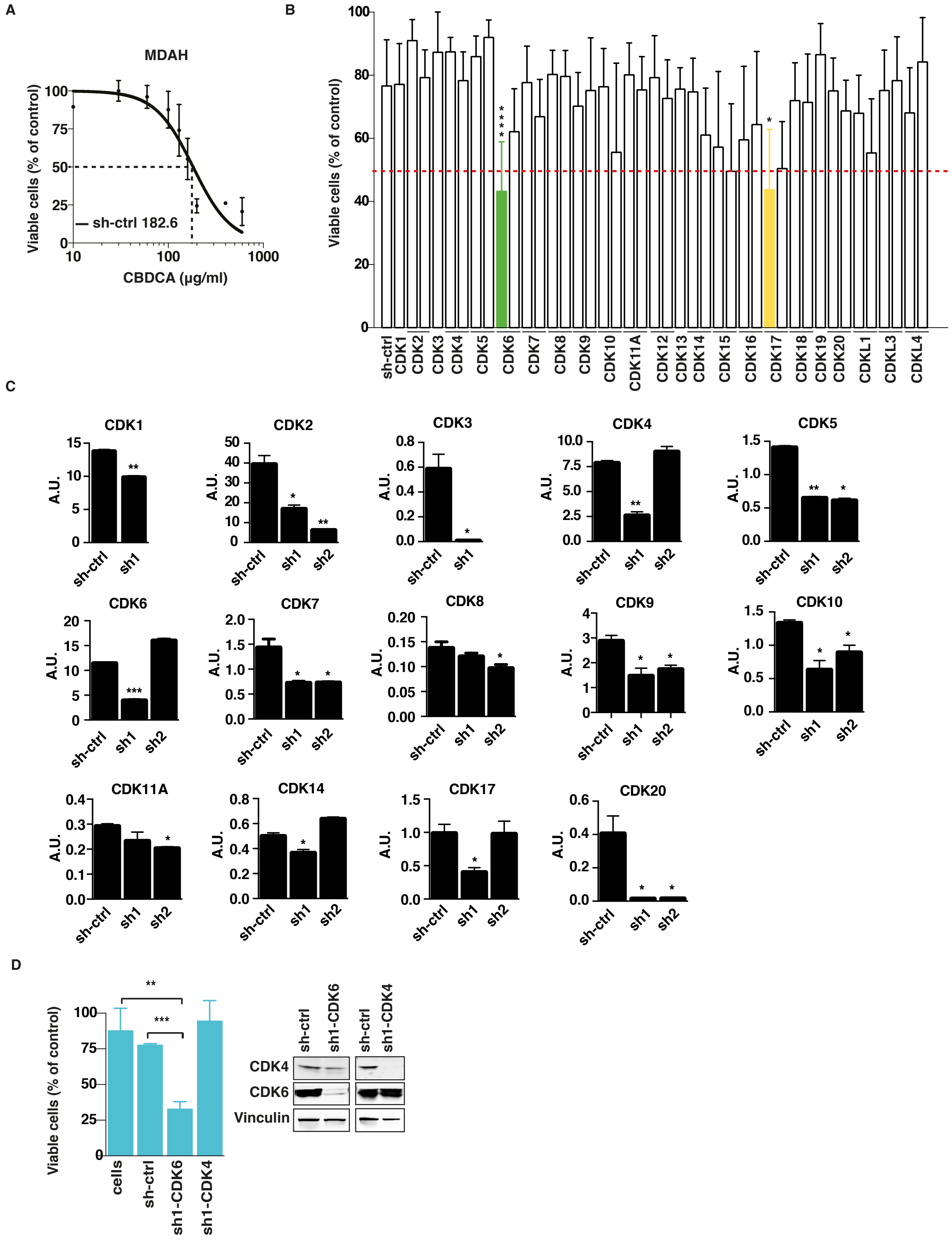

Appendix Figure S1

A

IC50 (μg/ml)	SKOV3ip sh-ctrl	SKOV3ip sh-CDK6
CDDP	33.8	16.8
CBDCA	120.5	69.4

B

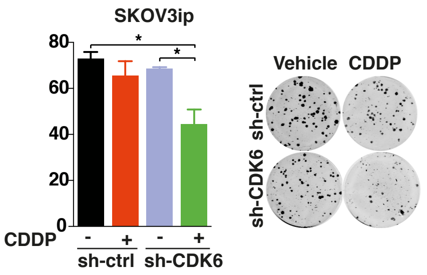

C

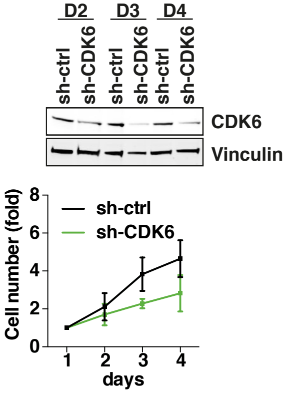

D

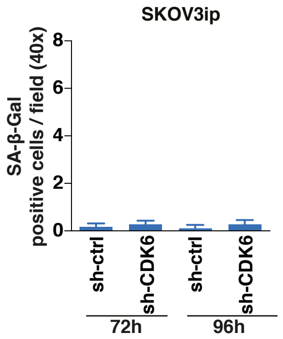

Appendix Figure S2

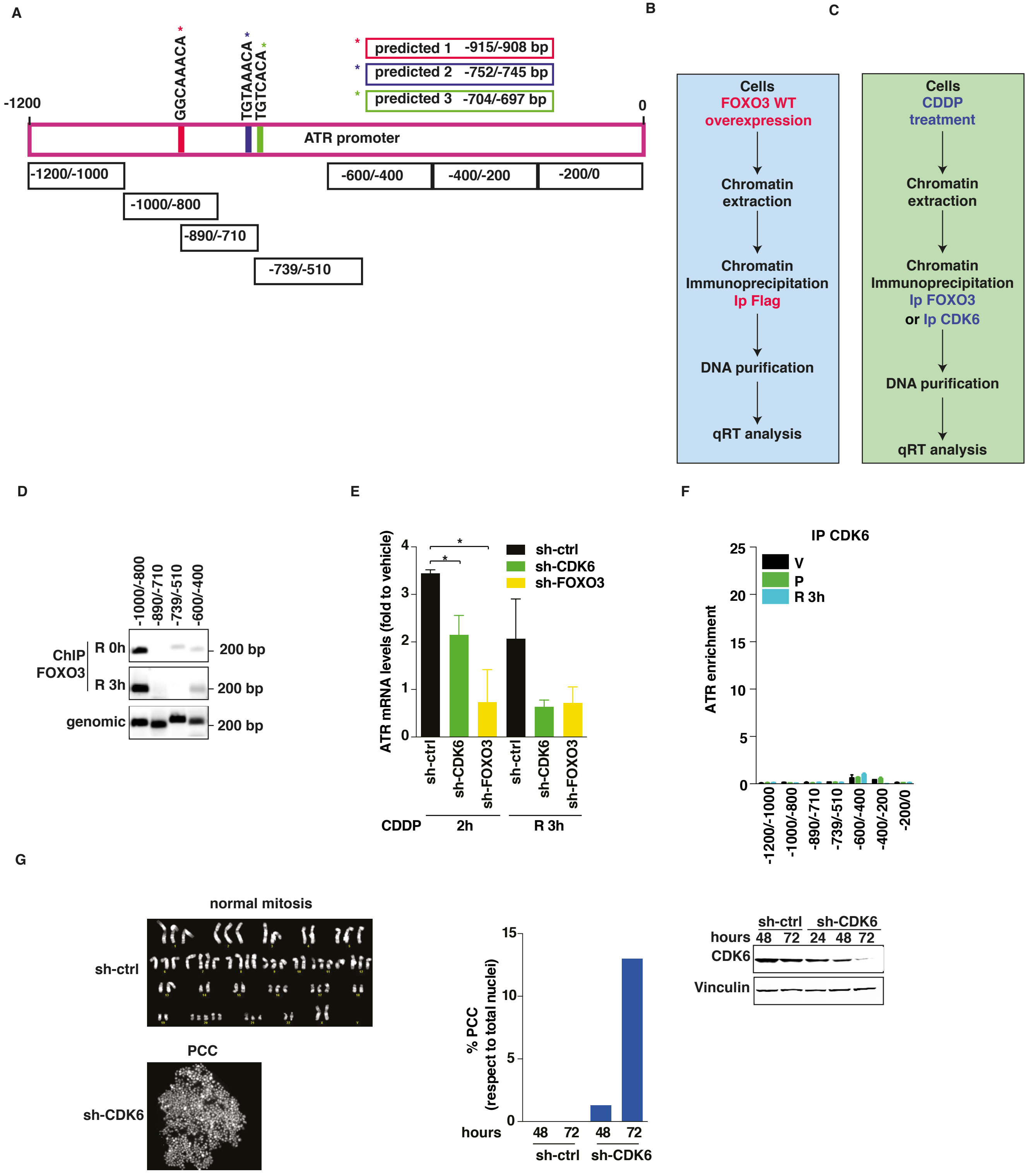

Appendix Figure S3

A

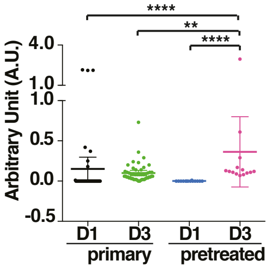

B

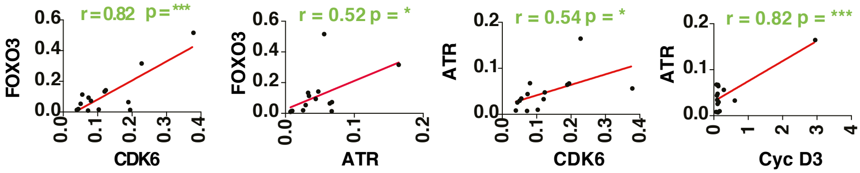

C

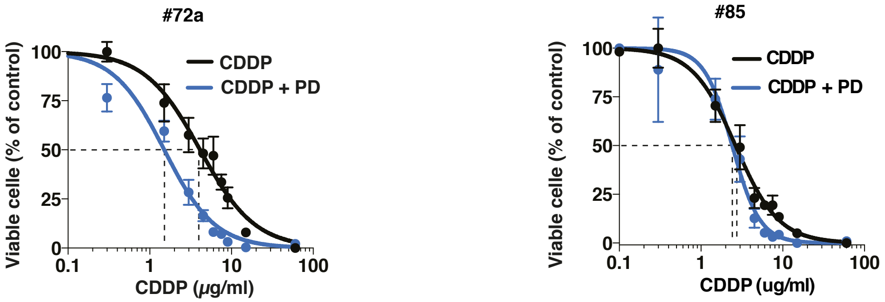

	CDDP	CDDP+PD
CDDP IC50	4.1	1.5

	CDDP	CDDP+PD
CDDP IC50	2.6	2.4

D

		univariate			multivariate		
		HR	95% CI	p value	HR	95% CI	p value
High Grade cases	CDK6 High vs Low	1.70	1.20-2.39	0.0025	1.61	1.12-2.32	0.010
	Stage III-IV vs I-II	7.57	2.80-20.49	< 0.0001	5.54	2.02-15.19	0.0009
	SD SOD vs OD	1.92	1.39-2.66	< 0.0001	1.58	1.13-2.19	0.0066

E

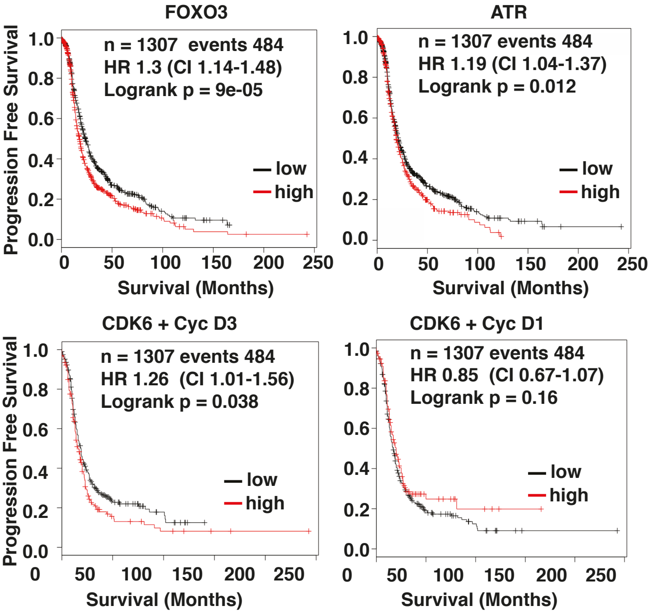

Appendix Figure S4
