# Supplementary material for: CDK6 protects epithelial ovarian cancer from platinum‐induced death via FOXO3 regulation
Source: EMBO Mol Med. 2017 Aug 4;9(10):1415–33. doi: 10.15252/emmm.201607012 (PMC5623833; doi:10.15252/emmm.201607012)
Supplement: Supplementary file 2 — Expanded View Figures PDF [file EMMM-9-1415-s002.pdf]

Expanded View Figures

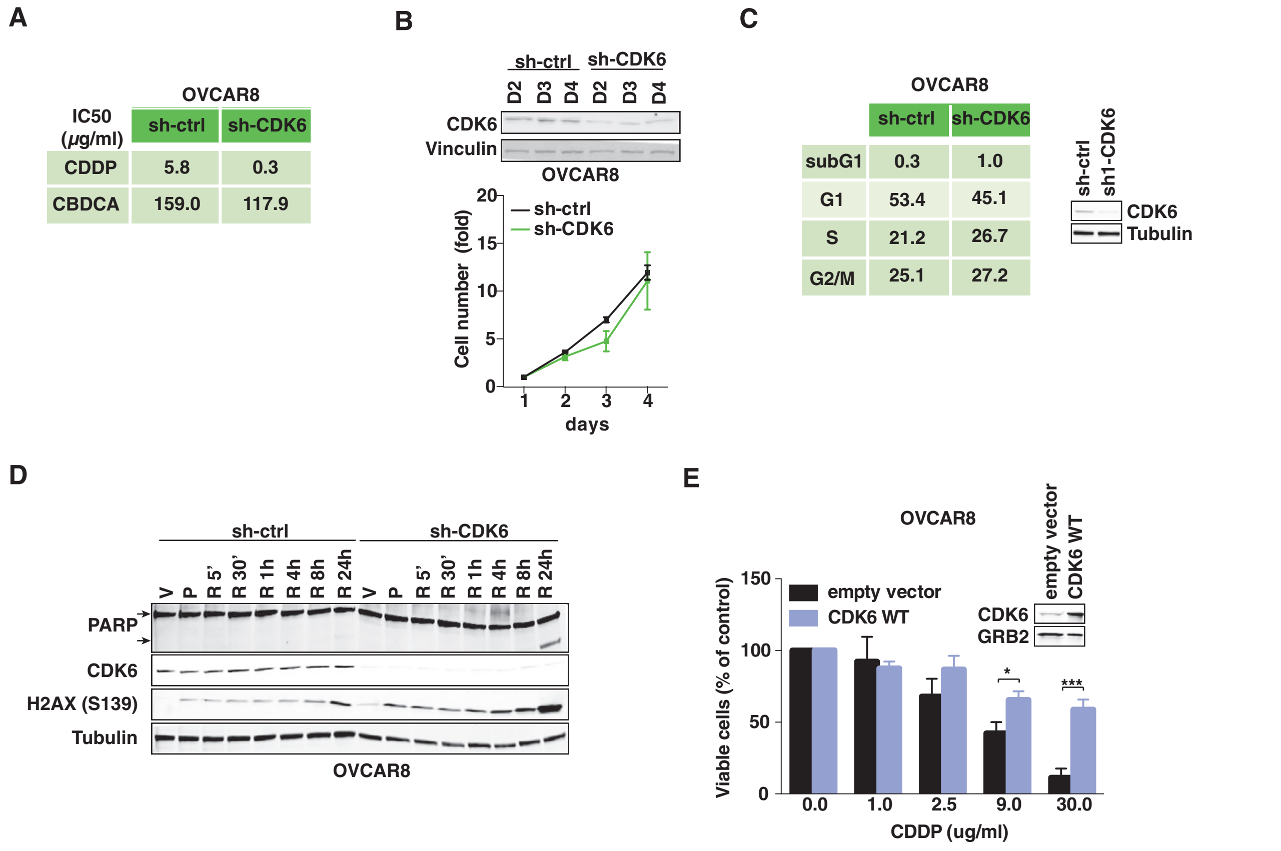

Figure EV1. CDK6 knockdown sensitizes OVCAR8 cells to platinum-induced cell death.

A Table summarizing CDDP and CBDCA IC50 of OVCAR8 cells transduced with ctrl or CDK6-specific shRNAs. Results are expressed as percentage of viable cells with respect to untreated cells and the resulting IC50 (half maximal inhibitory concentrations) are reported (n = 3 performed in triplicate).

B Growth curve of control or CDK6 silenced OVCAR8 cells. Data represent the mean ± SD of two biological replicates (each performed in triplicate). The corresponding cell lysates were analyzed by Western blot for CDK6 expression. Vinculin was used as loading control

C Cell cycle distribution of OVCAR8 cells transduced with control or CDK6-specific shRNAs evaluated by flow cytometry 72 h post-transduction. The corresponding cell lysates were analyzed by Western blot for CDK6 expression. Tubulin was used as loading control.

D Western blot evaluating PARP expression and cleavage and $\gamma H2AX^{S139}$ and CDK6 expression in OVCAR8 cells transduced with control or CDK6 shRNA and treated with 60 µg/ml of CBDCA for 16 h (P) and the released in platinum-free medium for the indicated times. V indicates control cells treated with vehicle. Tubulin was used as loading control.

E Cell viability of OVCAR8 cells transfected with CDK6 WT or empty vector and treated with increasing doses of CDDP for 16 h. Results are expressed as percentage of viable cells with respect to untreated cells. Data represent the mean ± SD of three biological replicates (two-sided, unpaired t-test). The corresponding cell lysates were analyzed by Western blot for CDK6 expression. GRB2 was used as loading control.

Data information: In each panel, significant differences are evidenced by asterisks (*P < 0.05, ***P < 0.001) and the exact P-values of (E) are reported in Appendix Table S4.
Source data are available online for this figure.

 © 2017 The Authors

Figure EV2. CDK6 kinase activity protects from platinum-induced EOC cell death *in vitro*.

A Expression of the indicated proteins in immortalized human epithelial ovarian cells (IHEOC) and in a panel of EOC-derived cell lines. Actin was used as loading controls.

B Cell viability of MDAH cells transfected with CDK6 WT, constitutively active (R31C), dominant negative (D163N), or empty vector (E.V.) and treated with 140 μg/ml of CBDCA for 16 h. Data represent the mean ± SD of two independent experiments performed in triplicate (two-sided, unpaired *t*-test).

C, D Dose–response curve of KURAMOCHI cells (C) treated with increasing doses of CDDP with or without the appropriate concentration of PD (i.e., 50% of the IC50) ($n = 2$). Data represent the mean ± SD of three biological replicates. The CDDP IC50 of parental KURAMOCHI, OVSAHO, and MDAH cells calculated as in (C) is reported in (D).

E MDAH cells transduced with the indicated shRNAs were treated with CDDP with or without PD (8 μM). Table reports the IC50 calculated as in (C). Data represent the mean of three biological replicates. On the top is reported the Western blot analysis evaluating the expression of CDK6 in control and silenced cells. Actin was used as loading control.

F Dose–response curve of parental MDAH cells and two different platinum-resistant cell clones treated with increasing doses of CDDP. The table reports the calculated IC50 of the three cell lines and represents the mean ± SD of six biological replicates.

G, H mRNA (G) and protein (H) expression of CDK6 in parental MDAH cells and two different platinum-resistant cell clones. In (G), data are expressed as normalized to housekeeping pol2A (two-sided, unpaired *t*-test). Error bars represent SD. In (H), GRB2 was used as loading control.

I Graph reporting the number of SA-β-galactosidase-positive cells/field (40× objective) in MDAH cells treated with PD and/or CBDCA as indicated for 72 h. Data represent the mean ± SD of two independent experiments, each performed in triplicate.

J Cell cycle distribution of OVCAR8 (left) and SKOV3ip (right) cells treated as indicated (PD 8 μM and CDDP 1.5 μg/ml) and the evaluated by flow cytometry.

Data information: In each panel, significant differences are evidenced by asterisks (*$P < 0.05$, **$P < 0.01$, ***$P < 0.001$) and the exact *P*-values of (B, G, and I) are reported in Appendix Table S4.

Source data are available online for this figure.

A

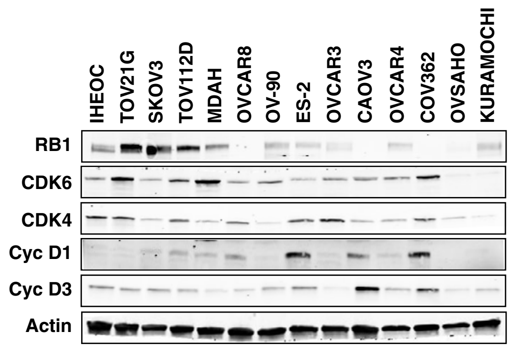

B

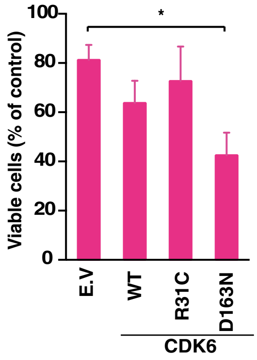

C

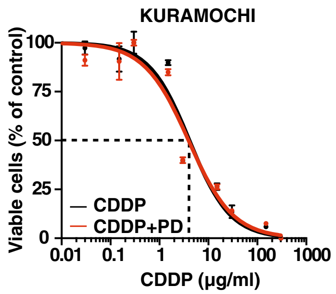

D

IC50 µg/ml	CDDP	CDDP +PD
MDAH	6.2	2.5
OVSAHO	1.3	1.2
KURAMOCHI	4.1	3.9

E

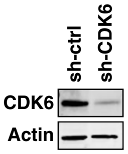

IC50 µg/ml	CDDP	CDDP +PD
MDAH sh-ctrl	8.4	4.3
MDAH sh-CDK6	5.1	4.6

F

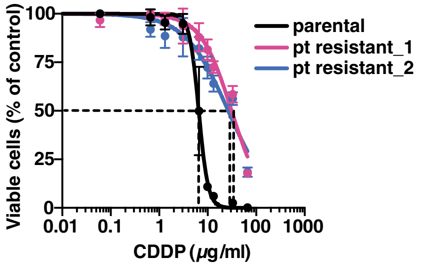

	parental	pt resistant_1	pt resistant_2
CDDP IC50	6.5	31.2	26.6

G

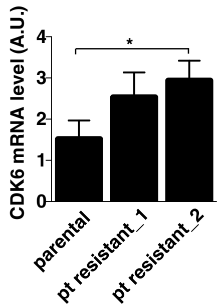

H

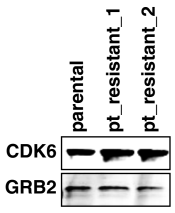

I

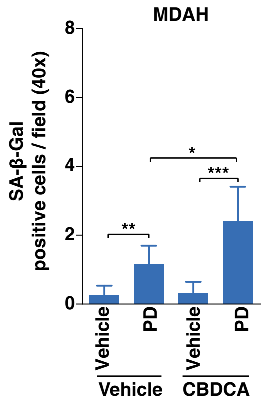

J

	Vehicle	PD 24h	CDDP 16h	CDDP 16h (R 24h)	CDDP 16h + PD 24h	CDDP 16h + PD 24h (R 24h)
sub G1	0.3	0.9	1.4	1.7	2.8	5.6
G1	42.0	39.1	25.6	22.3	10.6	32.6
S	26.3	28.4	60.7	22.5	14.4	26.3
G2/M	31.4	31.6	12.3	53.5	72.2	35.5

OVCAR8

	Vehicle	PD 24h	CDDP 16h	CDDP 16h (R 24h)	CDDP 16h + PD 24h	CDDP 16h + PD 24h (R 24h)
sub G1	0.3	5.7	0.9	5.2	4.9	6.9
G1	41.5	67.3	37.3	24.7	31.5	41.5
S	45.5	12.6	40.6	63.6	57.3	44.6
G2/M	12.7	14.4	21.2	6.5	6.3	7.0

SKOV3ip

Figure EV2.

© 2017 The Authors

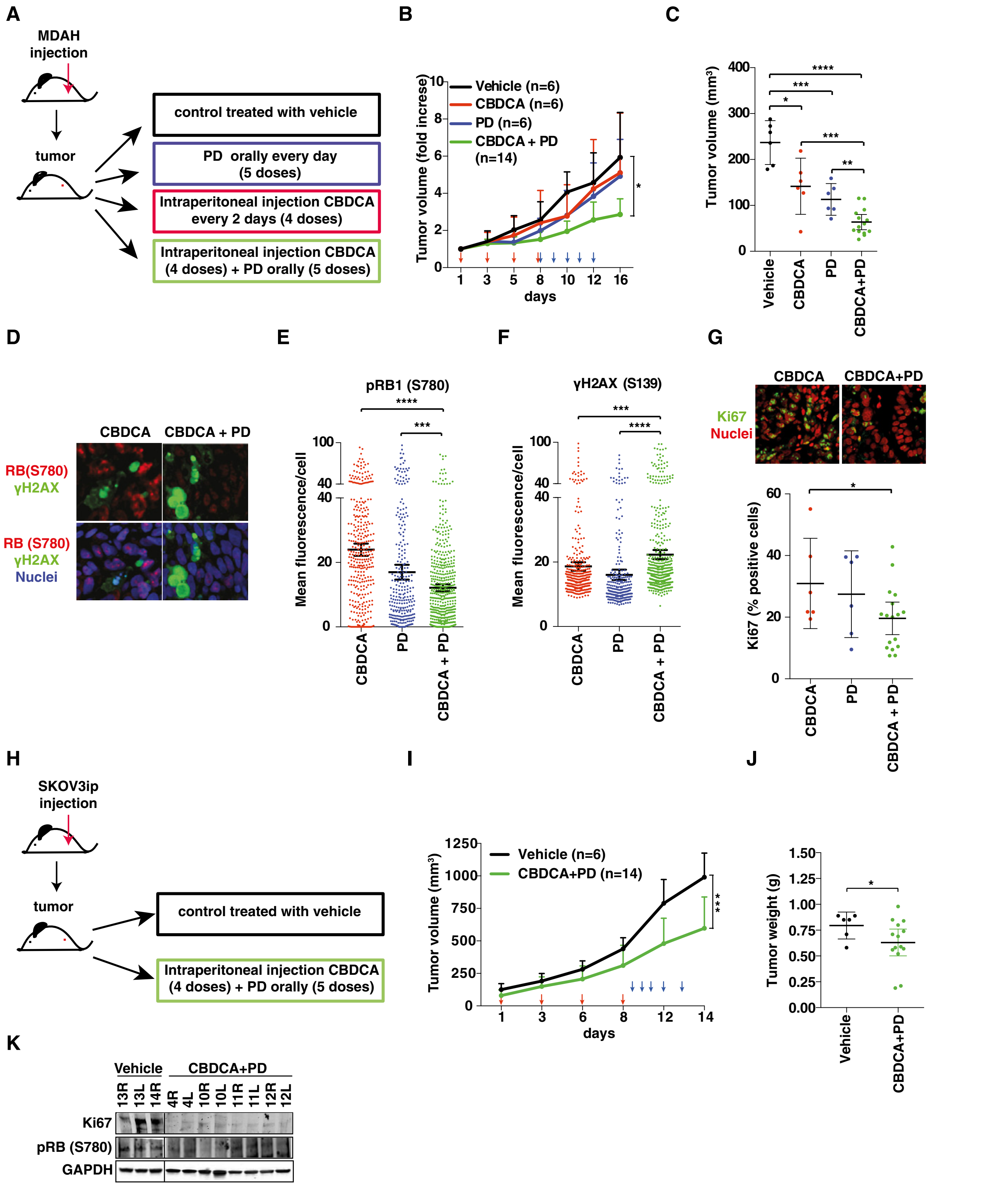
A
MDAH injection
tumor
control treated with vehicle
PD orally every day (5 doses)
Intraperitoneal injection CBDCA every 2 days (4 doses)
Intraperitoneal injection CBDCA (4 doses) + PD orally (5 doses)
B
Vehicle (n=6)
CBDCA (n=6)
PD (n=6)
CBDCA + PD (n=14)
Tumor volume (fold increse)
days
C
Tumor volume (mm³)
Vehicle
CBDCA
PD
CBDCA+PD
D
CBDCA
CBDCA + PD
RB(S780)
γH2AX
RB (S780)
γH2AX
Nuclei
E
pRB1 (S780)
Mean fluorescence/cell
CBDCA
PD
CBDCA + PD
F
γH2AX (S139)
Mean fluorescence/cell
G
CBDCA
CBDCA+PD
Ki67
Nuclei
Ki67 (% positive cells)
H
SKOV3ip injection
tumor
control treated with vehicle
Intraperitoneal injection CBDCA (4 doses) + PD orally (5 doses)
I
Vehicle (n=6)
CBDCA+PD (n=14)
Tumor volume (mm³)
days
J
Tumor weight (g)
Vehicle
CBDCA+PD
K
Vehicle
CBDCA+PD
13R 13L 14R 4R 4L 10R 10L 11R 11L 12R 12L
Ki67
pRB (S780)
GAPDH

Figure EV3.

◀ **Figure EV3. CDK6 protects EOC cells from platinum-induced death *in vivo*.**

A Schematic design of *in vivo* experiments with MDAH xenografts testing the efficacy of suboptimal doses of CBDCA (20 mg/kg) and PD (150 mg/kg) alone and in combination.

B, C Analysis of tumor growth (B) and tumor volume (C) of the experiment described in (A). CBDCA and PD treatments were indicated with red and blue arrows respectively. Analyzed tumors in each group are indicated in the graphs (two-sided, unpaired *t*-test). In (B) error bars represent SD. In (C), bars indicate mean ± 95% CI (Mann–Whitney test). In (C), each dot represents one mice.

D IF analyses of $pRB1^{S780}$ and $\gamma H2AX^{S139}$ in tumors explanted from mice treated with CBDCA or with CBDCA + PD.

E–G Quantification of $pRB1^{S780}$ (E), $\gamma H2AX^{S139}$ (F), and Ki67 (G) expression in explanted tumors (n = 3 per group) in which at least three randomly selected fields were studied) treated as indicated and evaluated as mean fluorescence per cell (E and F) or as percentage of positive cells (G). Bars indicate mean ± 95% CI (Mann–Whitney test). Panel (G) also shows IF analyses of Ki67 expression in explanted tumors.

H Schematic design of *in vivo* experiments with SKOV3ip xenografts testing the efficacy of suboptimal doses of CBDCA (20 mg/kg) and PD (150 mg/kg) in combination.

I, J Analysis of tumor growth (I) and tumor volume (J) of the experiment described in (H). The number of analyzed tumors in each group is reported in the graphs (two-sided, unpaired *t*-test). In (I) error bars indicate SD and in (J), bars indicate the mean ± 95% CI (Mann–Whitney test). Timing of CBDCA and PD treatments is reported in (I) and indicated with red and blue arrows, respectively. In (J) each dot represents one tumor.

K Western blot analyses evaluating the expression of $pRB1^{S780}$ and Ki67 in tumors explanted from mice treated with vehicle or CBDCA + PD described in (H). GAPDH was used as loading control.

Data information: In each panel, significant differences are evidenced by asterisks (*$P < 0.05$, **$P < 0.01$, ***$P < 0.001$, ****$P < 0.0001$) and the exact *P*-values of (B, C, E–G, I, and J) are reported in Appendix Table S4.

Source data are available online for this figure.

 © 2017 The Authors

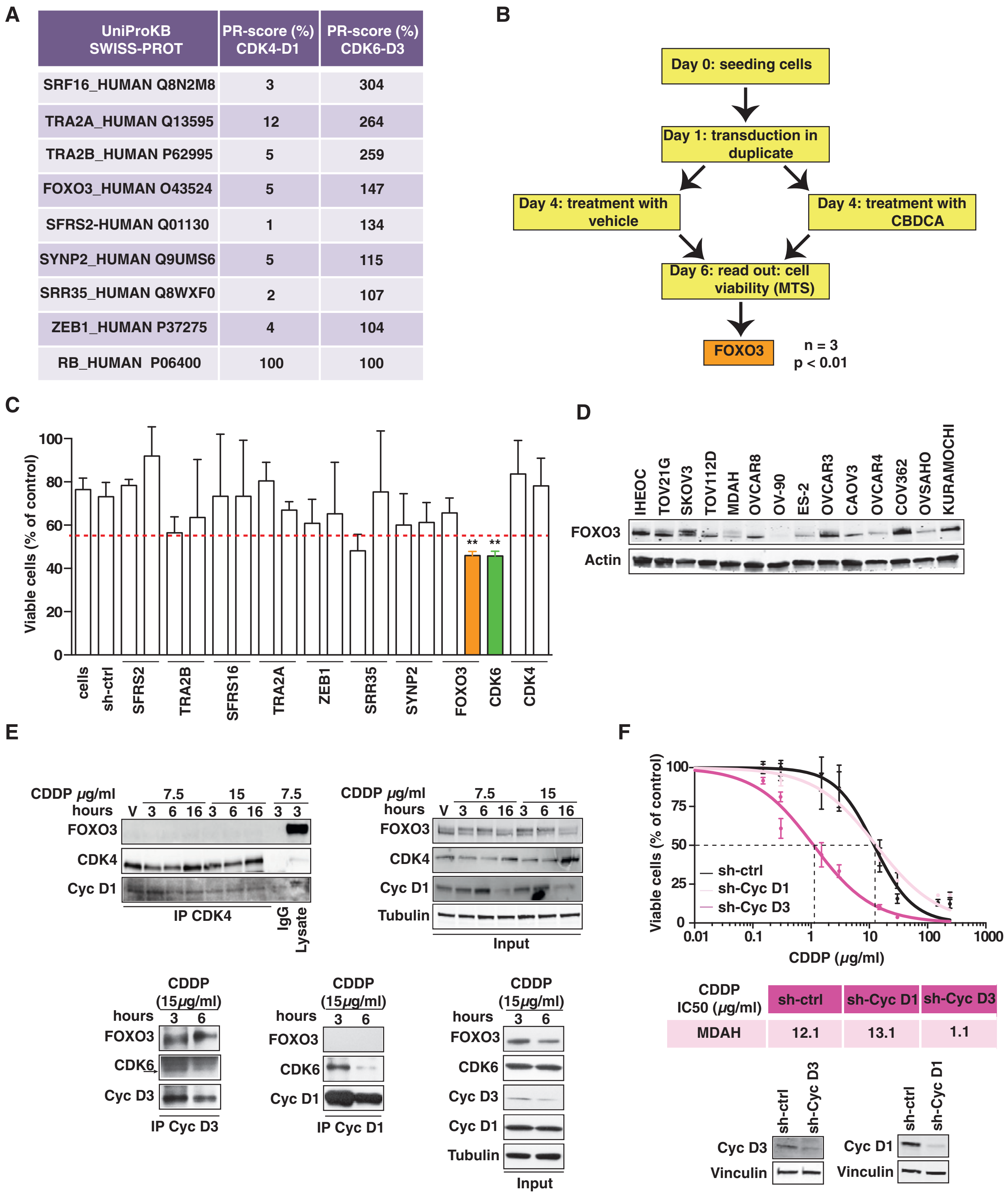

UniProKB SWISS-PROT	PR-score (%) CDK4-D1	PR-score (%) CDK6-D3
SRF16_HUMAN Q8N2M8	3	304
TRA2A_HUMAN Q13595	12	264
TRA2B_HUMAN P62995	5	259
FOXO3_HUMAN O43524	5	147
SFRS2-HUMAN Q01130	1	134
SYNP2_HUMAN Q9UMS6	5	115
SRR35_HUMAN Q8WXF0	2	107
ZEB1_HUMAN P37275	4	104
RB_HUMAN P06400	100	100

CDDP IC50 (μg/ml)	sh-ctrl	sh-Cyc D1	sh-Cyc D3
MDAH	12.1	13.1	1.1

Figure EV4.

◀ **Figure EV4. FOXO3 is a CDK6 phosphorylation target that controls platinum sensitivity in EOC cells.**

A Table reporting CDK6-specific phosphorylation targets ordered by their PR score [adapted from Anders *et al* (2011)]. The PR score represents the normalized phosphorylation levels of the indicated protein with respect to RB used as positive control (PR = 100).

B Experimental design of the loss-of-function screening performed on MDAH cells to evaluate the effect of silencing CDK6 phosphorylation targets. "*n*" indicates the number of independent experiments performed. Significance (*P*) was calculated by two-sided, unpaired *t*-test.

C Graph reporting the results of the loss-of-function screening described in (B). Data represent the mean ± SD of three independent experiments each performed in triplicate and graphed as survival ratio between of CBDCA-treated (140 μg/ml for 16 h) and untreated cells. Significance was calculated using two-sided, unpaired *t*-test. Significant differences are evidenced by asterisks (**$P < 0.01$), and the exact *P*-values reported in Appendix Table S4.

D Western blot analysis reporting the expression of FOXO3 in the indicated EOC cell lines. Actin was used as loading control.

E Co-IP analysis of endogenous CDK4 with FOXO3 and cyclin D1 (upper left panel) in MDAH cells treated with CDDP 7.5 or 15 μg/ml for the indicated times. In the right panels, expression of the same proteins in the corresponding lysates (Input) is reported. In the lower left panels are reported the Co-IP analyses of cyclin D1 and cyclin D3 with FOXO3 and CDK6 in cells treated for 3 or 6 h with 15 μg/ml of CDDP. In the right lower panels is reported the expression of the same proteins in the corresponding lysates (Input). Tubulin was used as loading control.

F Dose–response curve on MDAH cells transduced with control, cyclin D1, or cyclin D3 shRNAs and treated with increasing doses of CDDP for 16 h (three biological replicates). The resulted CDDP IC50 is reported in the table. Lower panels show the Western blot analyses of the expression of cyclins D1 and D3 in the transduced cells. Vinculin was used as loading control. Error bars represent SD.

Source data are available online for this figure.

 © 2017 The Authors

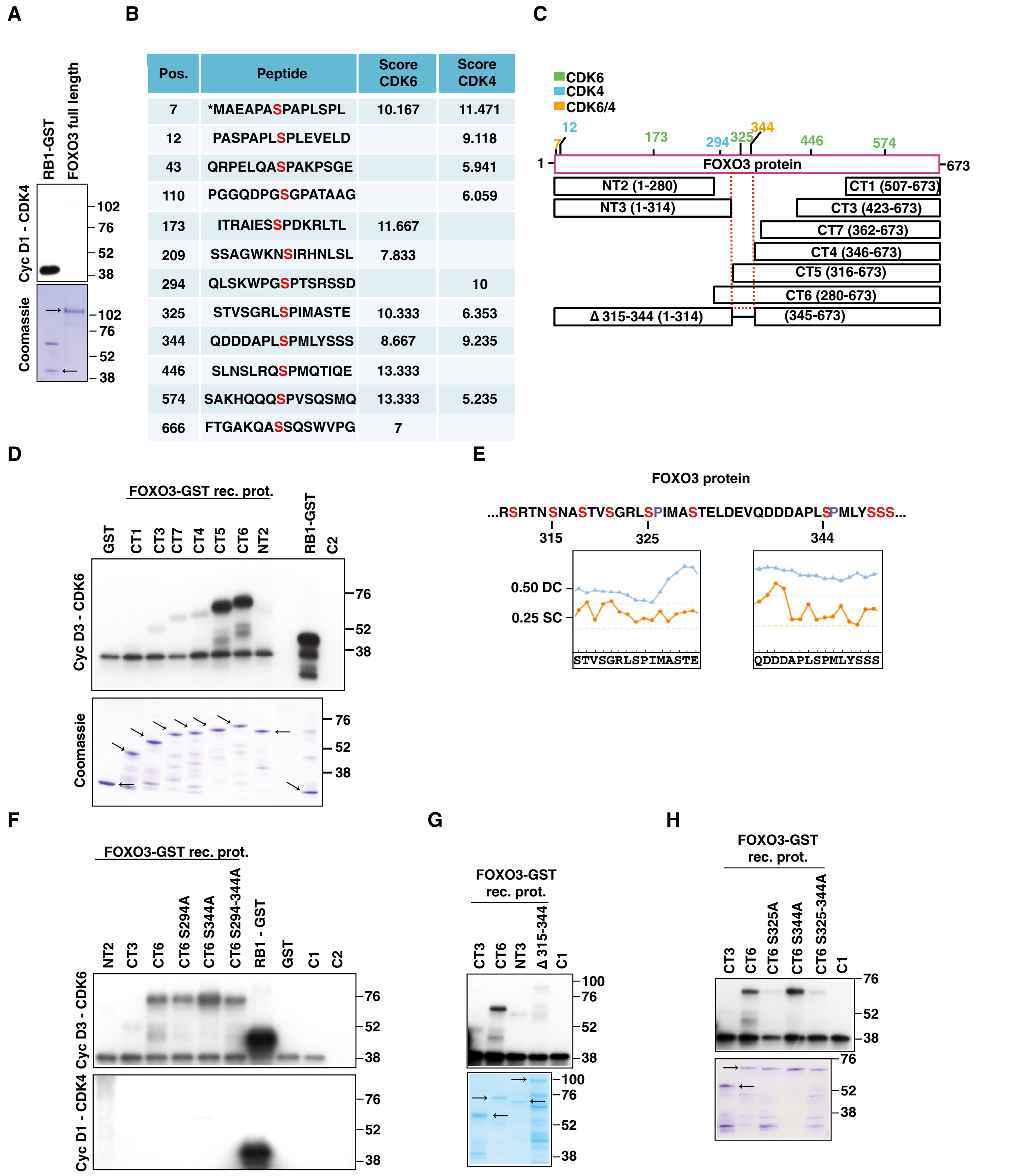

Pos.	Peptide	Score CDK6	Score CDK4
7	*MAEAPASPAPLSPL	10.167	11.471
12	PASPAPLSPLEVELD		9.118
43	QRPELQASPAKPSGE		5.941
110	PGGQDPGSGPATAAG		6.059
173	ITRAIESSPDKRLTL	11.667	
209	SSAGWKNSIRHNLSL	7.833	
294	QLSKWPGSPTSRSSD		10
325	STVSGRLSPIMASTE	10.333	6.353
344	QDDDAPLSPMLYSSS	8.667	9.235
446	SLNSLRQSPMQTIQE	13.333	
574	SAKHQQQSPVSQSMQ	13.333	5.235
666	FTGAKQASSQSWVPG	7	

Figure EV5.

Figure EV5. FOXO3 is phosphorylated by cyclin D3/CDK6 complex on S325.

A *In vitro* phosphorylation assay performed using recombinant cyclin D1-CDK4 complex and GST-RB1 fragment or FOXO3 full length as substrates. The Coomassie staining of gel loaded with the same amount of recombinant proteins used in the kinase assay is shown. Arrows indicate the expected molecular weight of GST and FOXO3 recombinant proteins.

B *In silico* analysis performed using the GPS3.0 prediction software available online (http://gps.biocuckoo.org/) of potentially phosphorylated sites on FOXO3 by CDK6/4. Pos. indicates the position of the phosphorylated serine. Peptide reports the sequence of the peptide containing the predicted phosphorylated serine (in red). The score indicates the probability of CDK6 or CDK4 to phosphorylate each indicated residue. The higher the score, the higher is the probability of phosphorylation.

C Schematic representation of FOXO3 predicted phosphorylation sites (with a score > 8 for CDK6 and/or CDK4) and FOXO3 deletion mutants used in this study (NT: N-terminus, CT: C-terminus). Sites predicted to be phosphorylated by CDK6, CDK4, or both CDKs with a score higher than 9 are reported in green, light blue, and orange, respectively.

D *In vitro* phosphorylation assays performed using recombinant cyclin D3-CDK6 complex and the indicated FOXO3 deletion mutants or GST-RB1 fragment as substrates. The Coomassie staining of companion gel is reported. Arrows indicate expected molecular weight of recombinant proteins. C2 = kinase reaction mix control.

E Amino acid sequence of FOXO3 region (residues 318–351) phosphorylated by CDK6 *in vitro* as experimentally determined using FOXO3 deletion mutants. Plots report the disorder and the surface exposure of the two peptides containing S325 (left) and S344 (right). Location of amino acids above the disordered (0.50 DC, dotted blue line) and surface exposure cutoff (0.25 EC, dotted orange line) indicates whether they fall in an intrinsically disordered (blue line) and surface-exposed region (orange line). In the top part of the panel the highlighted residues S315 and S344 indicate the first and the last deleted amino acid in the D315-344 deletion mutant. 325 highlighted the serine phosphorylated by CDK6.

F *In vitro* phosphorylation assays performed using recombinant cyclin D3-CDK6 (upper panel) or cyclin D1-CDK4 (lower panel) complexes and the indicated FOXO3 deletion mutants or GST-RB1 fragment as substrates. C1 = kinase reaction mix plus recombinant cyclin D3-CDK6; C2 = kinase reaction mix control.

G *In vitro* phosphorylation assays performed using recombinant cyclin D3-CDK6 and the indicated FOXO3 deletion mutants fragment as substrates. The Coomassie staining of companion gel is reported. Arrows indicate expected molecular weight of recombinant proteins. C1 = kinase reaction mix plus recombinant cyclin D3-CDK6.

H *In vitro* phosphorylation assays performed using recombinant cyclin D3-CDK6 and, as substrates, the indicated FOXO3 deletion mutants with or without the substitution of serine 325 and/or 344 to alanine. The Coomassie staining of companion gel is reported. Arrows indicate expected molecular weight of recombinant proteins. C1 = kinase reaction mix plus recombinant cyclin D3-CDK6.

Source data are available online for this figure.

© 2017 The Authors
