# Supplementary figures and images for: CDK6 protects epithelial ovarian cancer from platinum‐induced death via FOXO3 regulation
Source: EMBO Mol Med. 2017 Aug 4;9(10):1415–33. doi: 10.15252/emmm.201607012 (PMC5623833; doi:10.15252/emmm.201607012)

Figure EV5 Data source

Figure EV5A

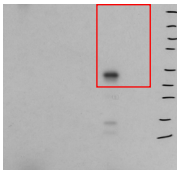

Figure EV5D

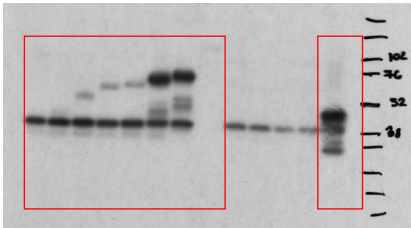

Figure EV5F

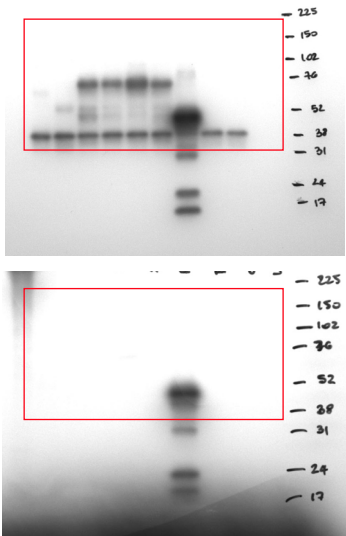

Figure EV5G

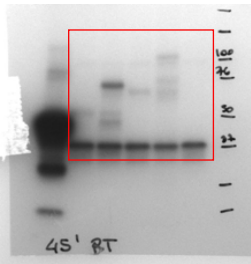

Figure EV5H

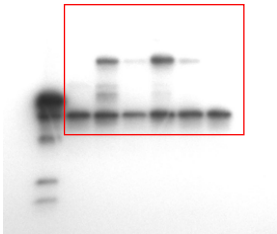

Supplement: Supplementary file 8 — Source Data for Expanded View and Appendix [file EMMM-9-1415-s015.zip › EMM_07012_EV_Appendix_SD/EMM_07012_SD_.EV5pdf.pdf]

Figure EV1 Data source

Figure EV1B

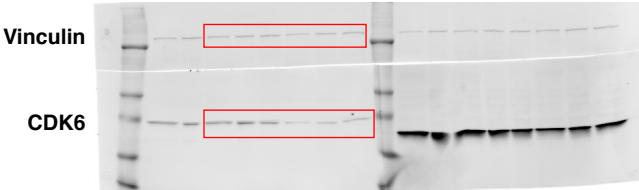

Figure EV1C

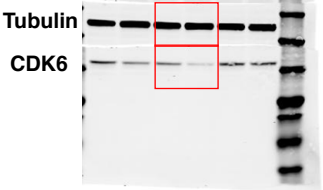

Figure EV1D

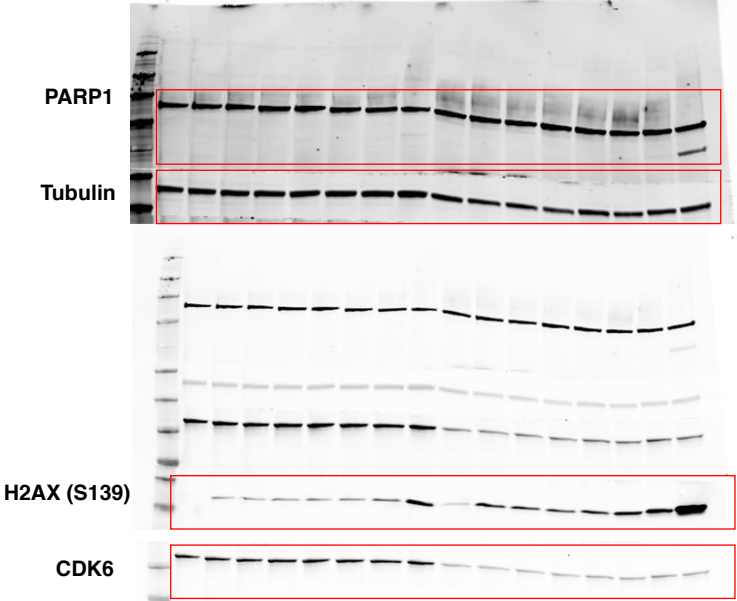

Figure EV1E

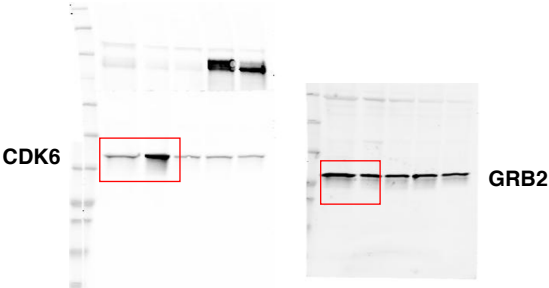

Supplement: Supplementary file 8 — Source Data for Expanded View and Appendix [file EMMM-9-1415-s015.zip › EMM_07012_EV_Appendix_SD/EMM_07012_SD_EV1.pdf]

## Figure EV2 Data source

Figure EV2A

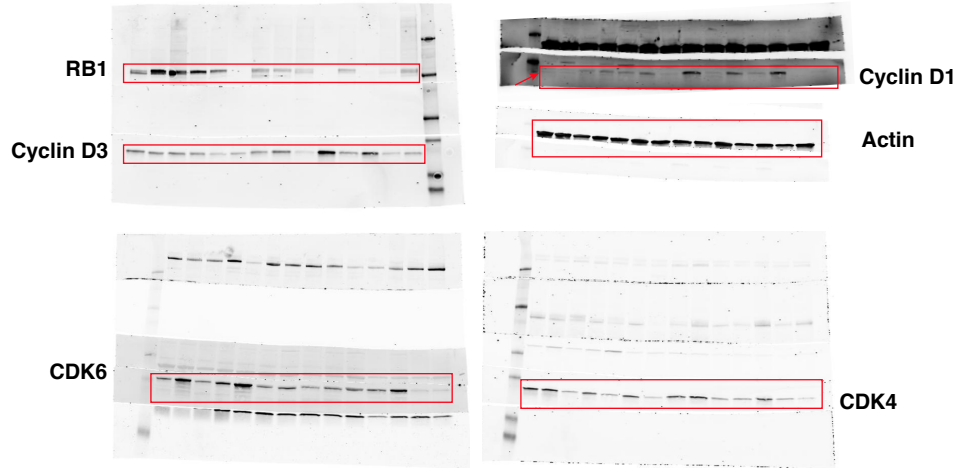

Figure EV2E

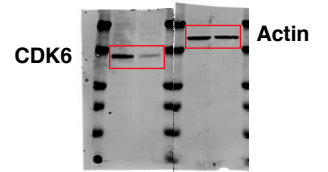

Figure EV2H

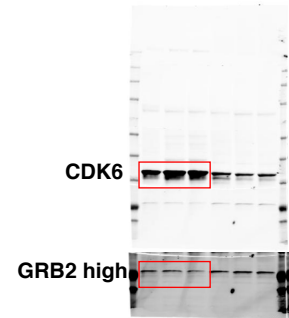

Supplement: Supplementary file 8 — Source Data for Expanded View and Appendix [file EMMM-9-1415-s015.zip › EMM_07012_EV_Appendix_SD/EMM_07012_SD_EV2.pdf]

# Figure EV3 Data source

Figure EV3K

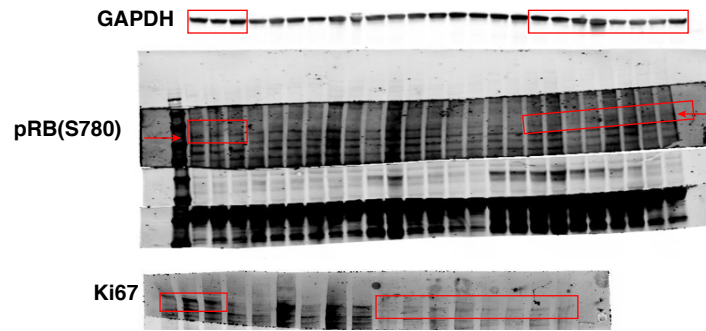

Supplement: Supplementary file 8 — Source Data for Expanded View and Appendix [file EMMM-9-1415-s015.zip › EMM_07012_EV_Appendix_SD/EMM_07012_SD_EV3.pdf]

## Figure EV4 Data source

Figure EV4D

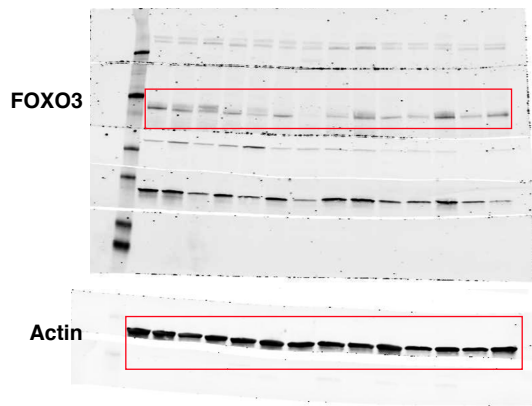

Figure EV4F

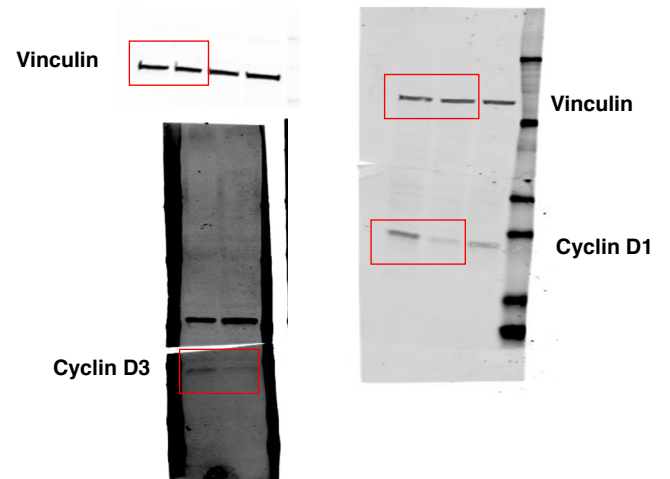

Figure EV4E

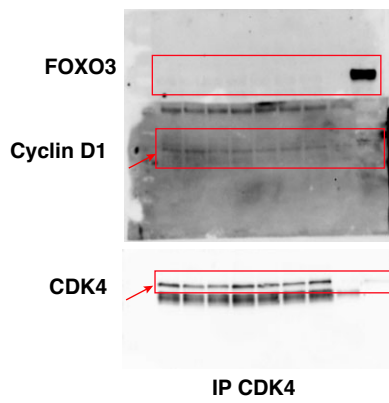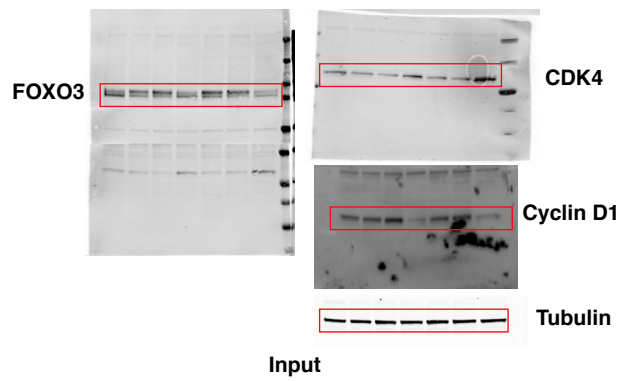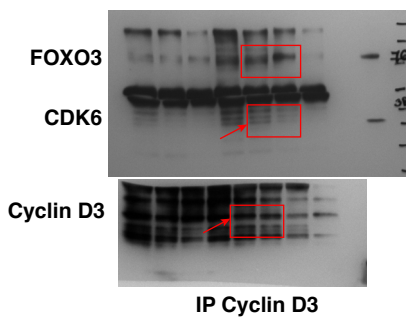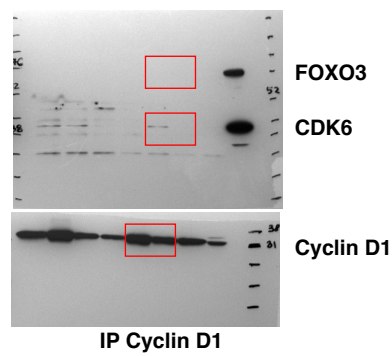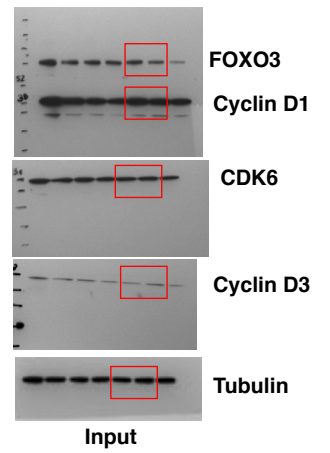

Supplement: Supplementary file 8 — Source Data for Expanded View and Appendix [file EMMM-9-1415-s015.zip › EMM_07012_EV_Appendix_SD/EMM_07012_SD_EV4.pdf]

Appendix Figure S1 Data source

Figure S1D

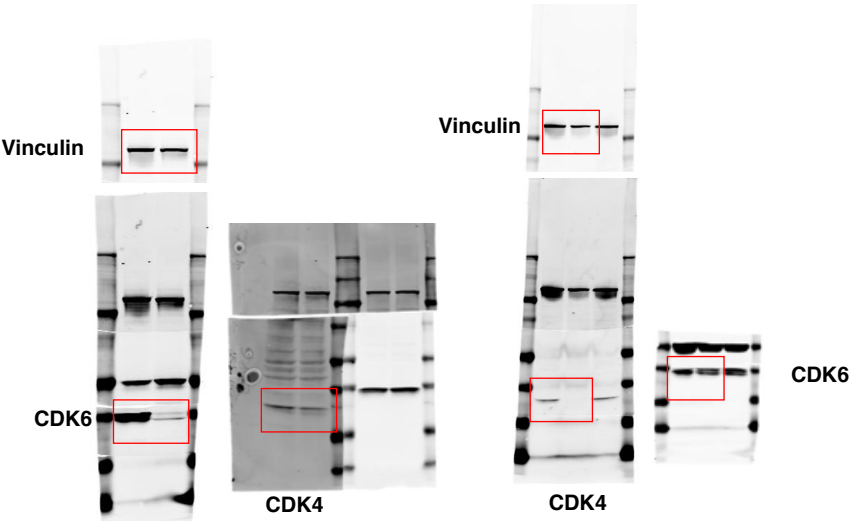

Supplement: Supplementary file 8 — Source Data for Expanded View and Appendix [file EMMM-9-1415-s015.zip › EMM_07012_EV_Appendix_SD/EMM_07012_SD_S1.pdf]

Appendix Figure S2 Data source

Figure S2C

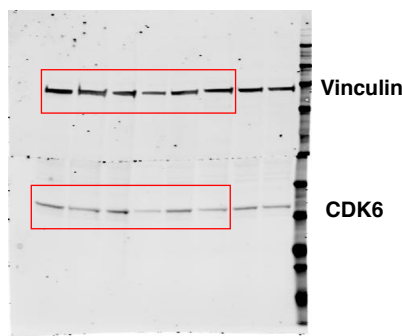

Supplement: Supplementary file 8 — Source Data for Expanded View and Appendix [file EMMM-9-1415-s015.zip › EMM_07012_EV_Appendix_SD/EMM_07012_SD_S2.pdf]

## Appendix Figure S3 Data source

Figure S3G

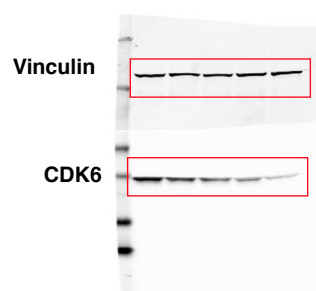

Supplement: Supplementary file 8 — Source Data for Expanded View and Appendix [file EMMM-9-1415-s015.zip › EMM_07012_EV_Appendix_SD/EMM_07012_SD_S3.pdf]

# Figure 1 Data source

Figure 1B

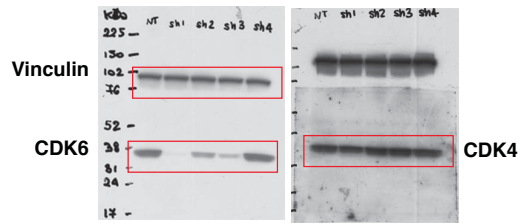

Figure 1E

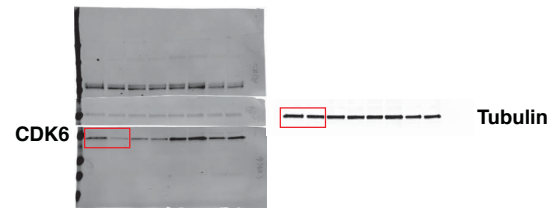

Figure 1F

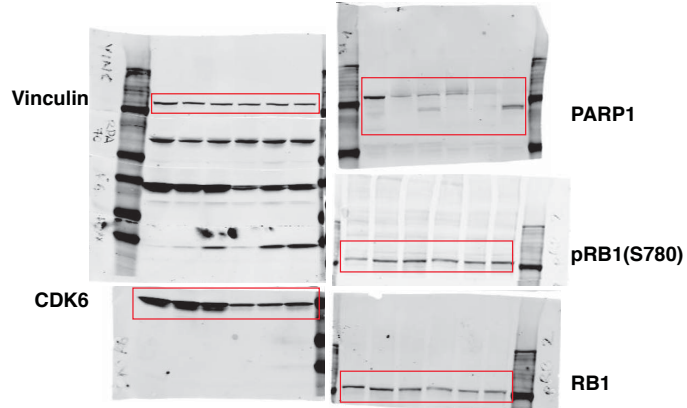

Figure 1G

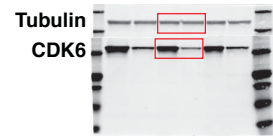

Figure 1I

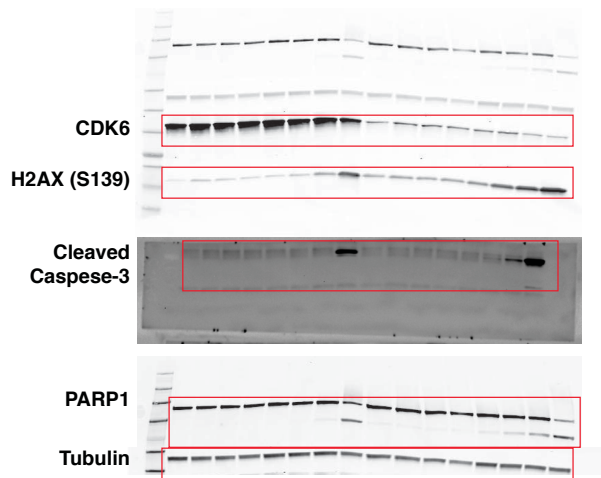

Supplement: Supplementary file 10 — Source Data for Figure 1 [file EMMM-9-1415-s008.pdf]

Figure 2 Data source

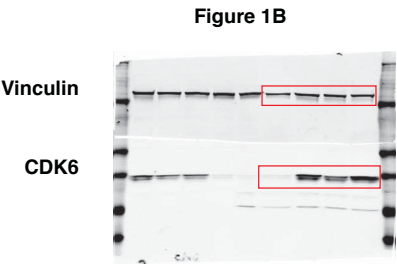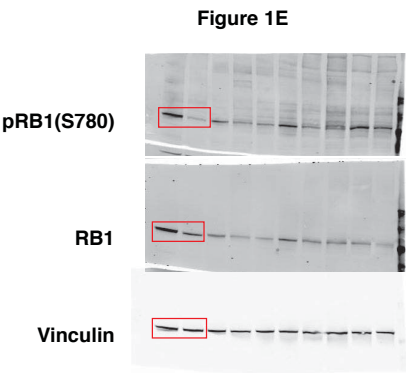

Supplement: Supplementary file 11 — Source Data for Figure 2 [file EMMM-9-1415-s009.pdf]

Figure 3 Data source

Figure 3D

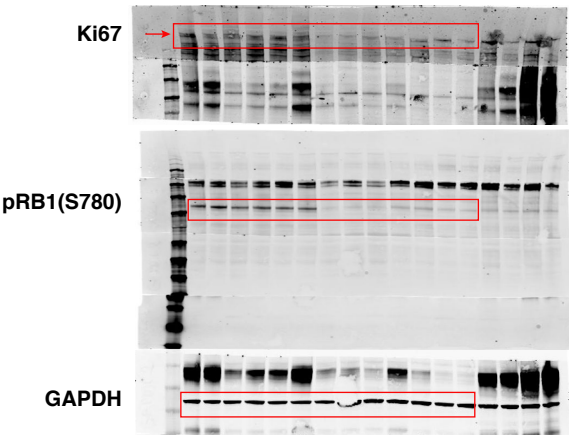

Figure 3H

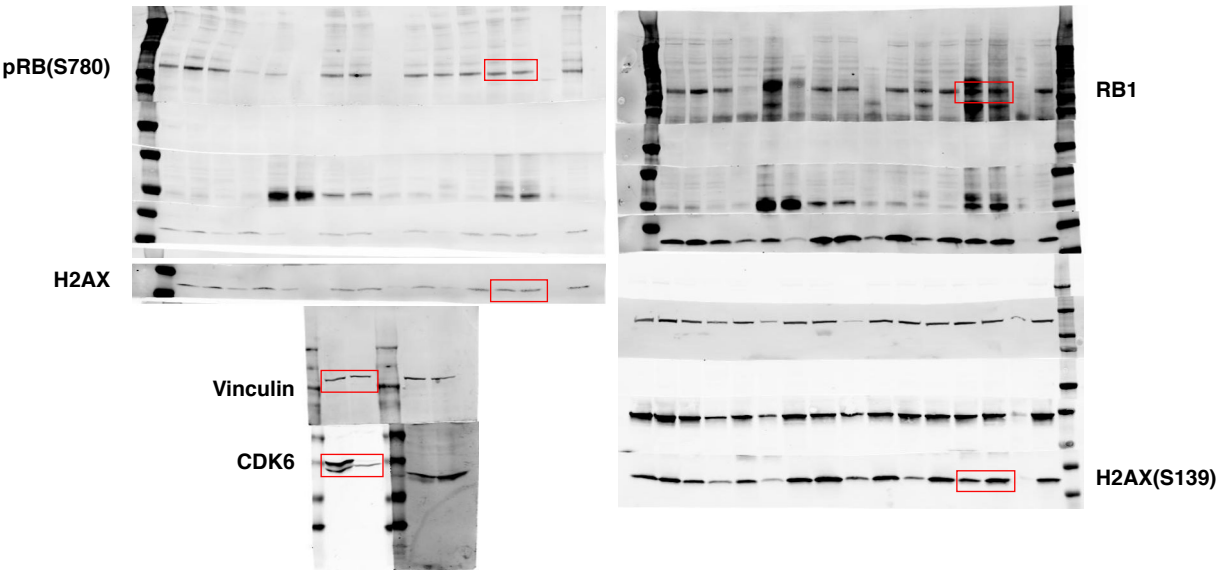

Supplement: Supplementary file 12 — Source Data for Figure 3 [file EMMM-9-1415-s010.pdf]

Figure 4 Data source

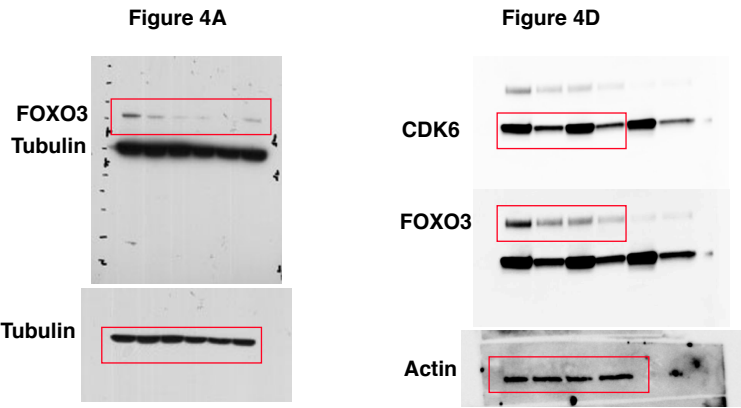

Figure 4E

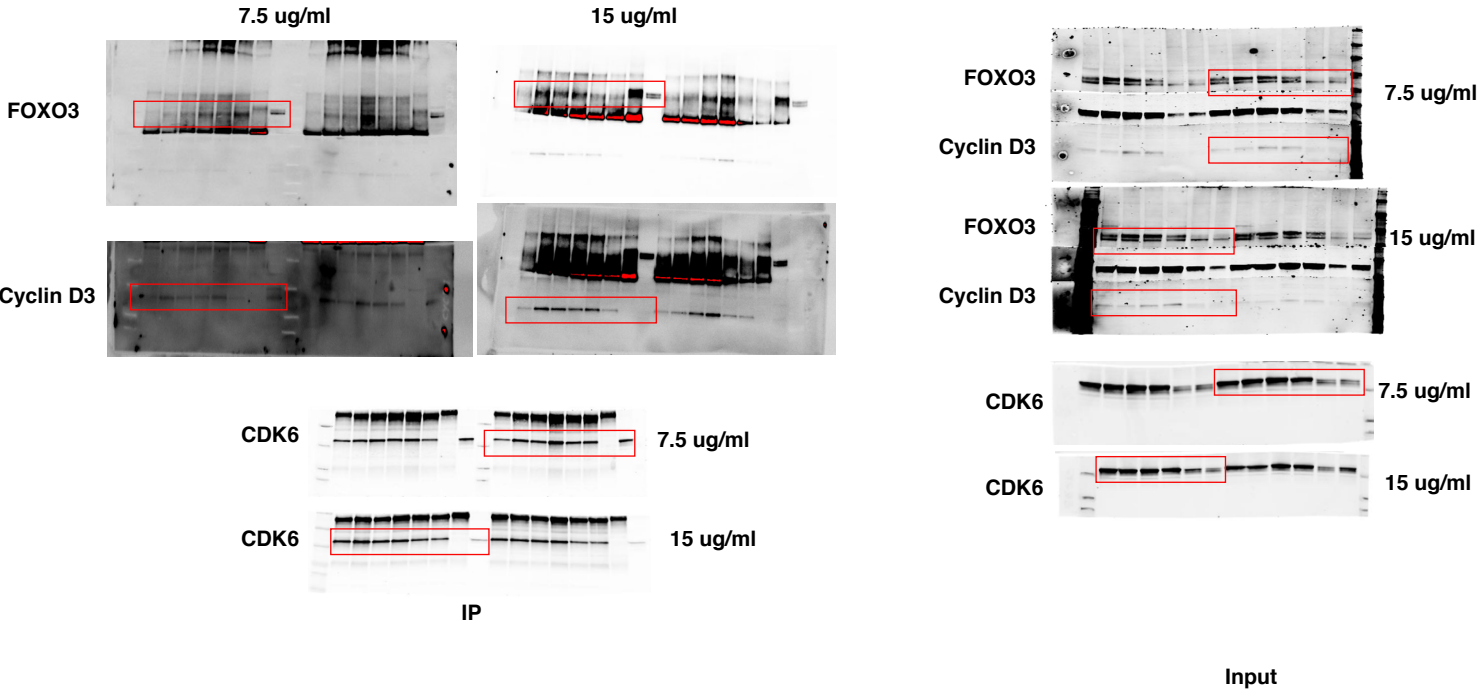

Figure 4F

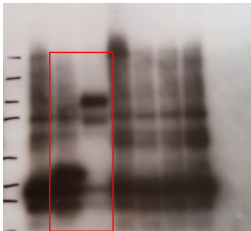

Figure 4G

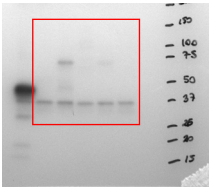

Figure H

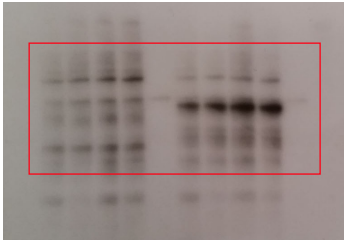

Supplement: Supplementary file 13 — Source Data for Figure 4 [file EMMM-9-1415-s011.pdf]

# Figure 5 Data source

Figure 5A

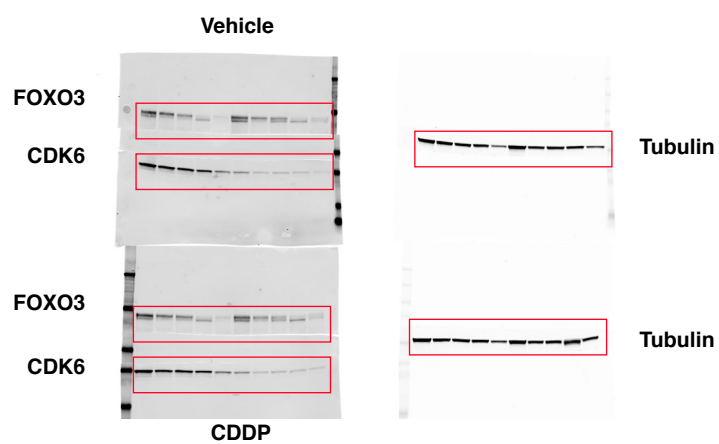

Figure 5B

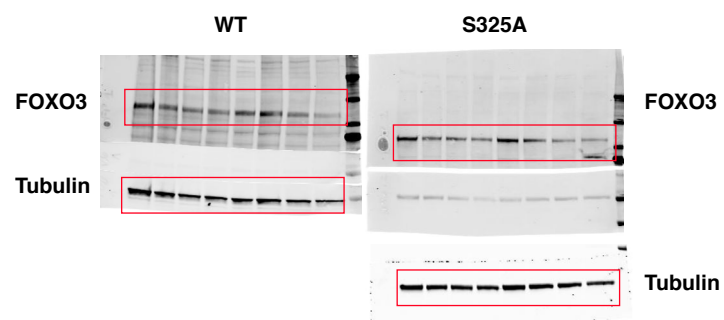

Figure 5C

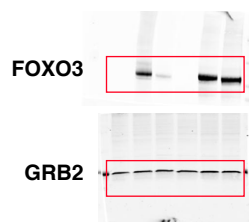

Figure 5G

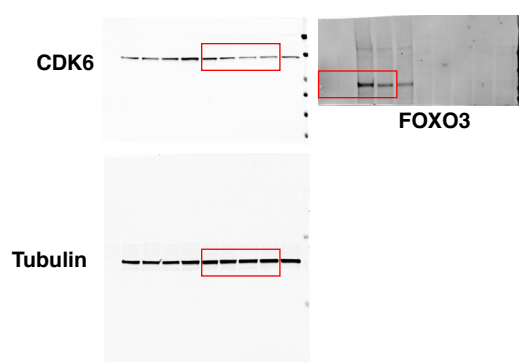

Figure 5H

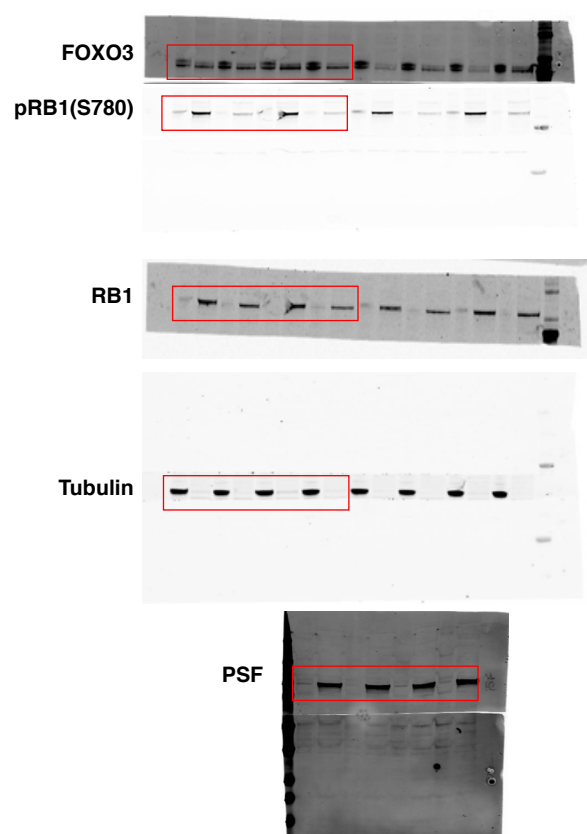

Supplement: Supplementary file 14 — Source Data for Figure 5 [file EMMM-9-1415-s012.pdf]

# Figure 6 Data source

Figure 6A

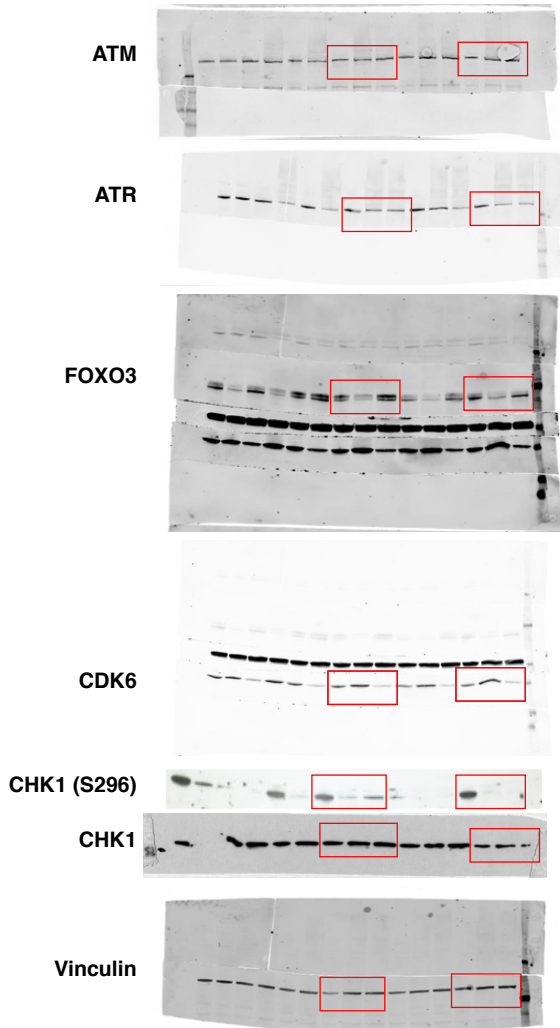

Figure 6B

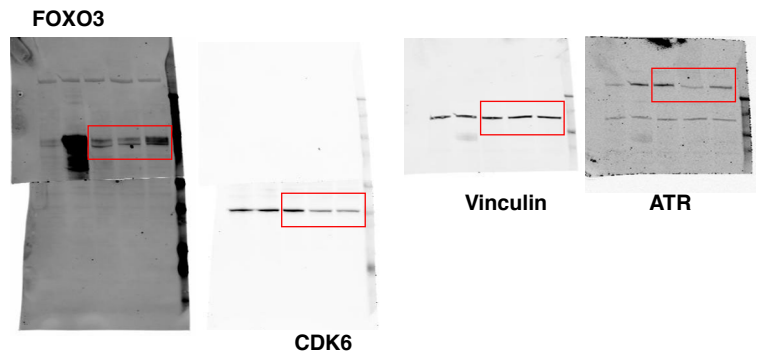

Figure 6C

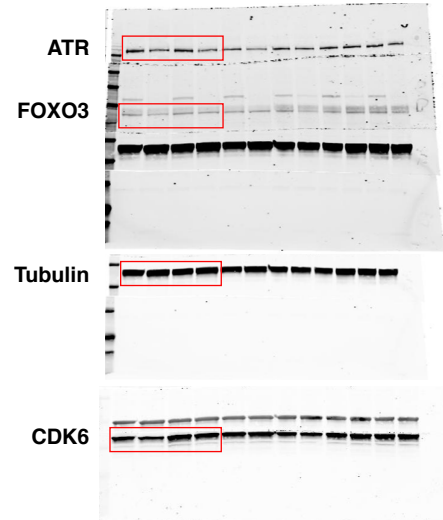

Figure 6D

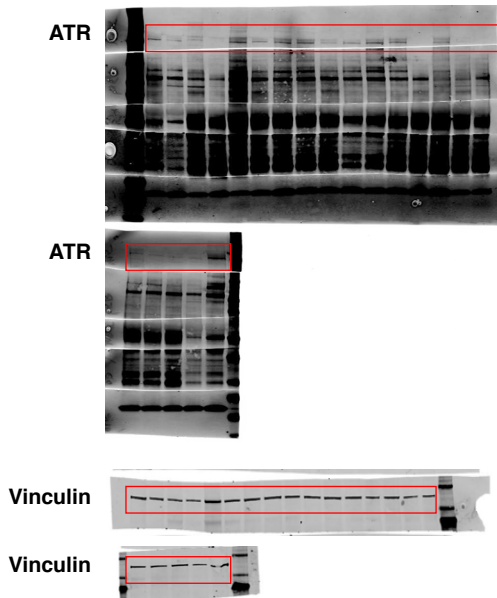

Figure 6G

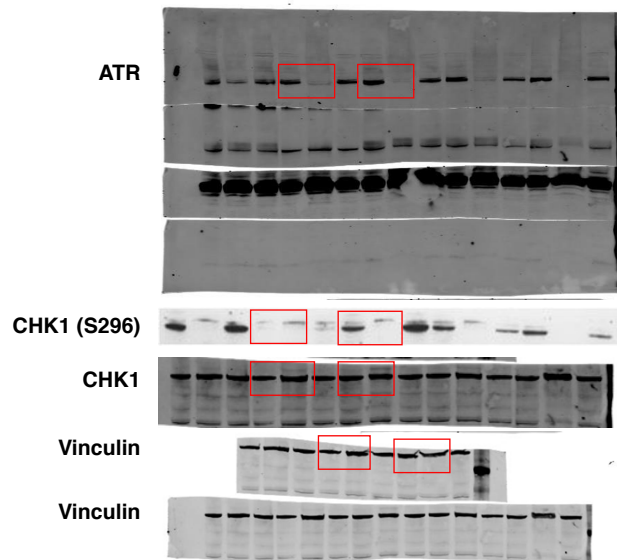

Supplement: Supplementary file 15 — Source Data for Figure 6 [file EMMM-9-1415-s013.pdf]

## Figure 7 Data source

Figure 7B

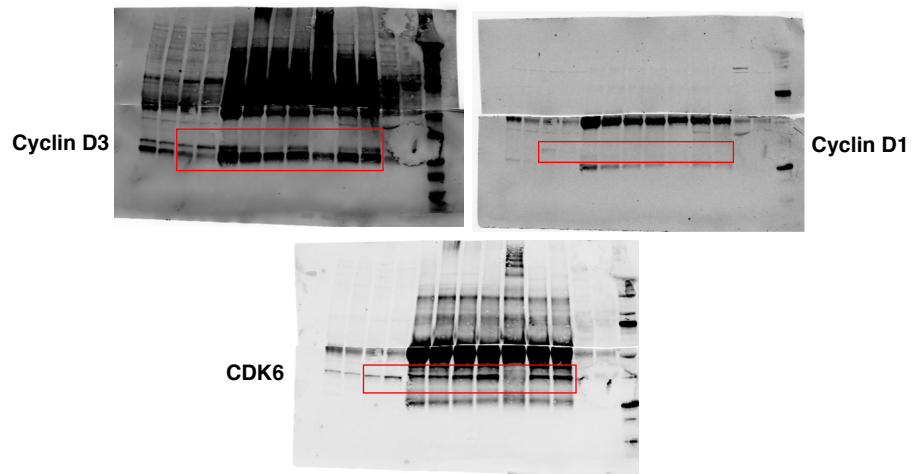

Figure 7C

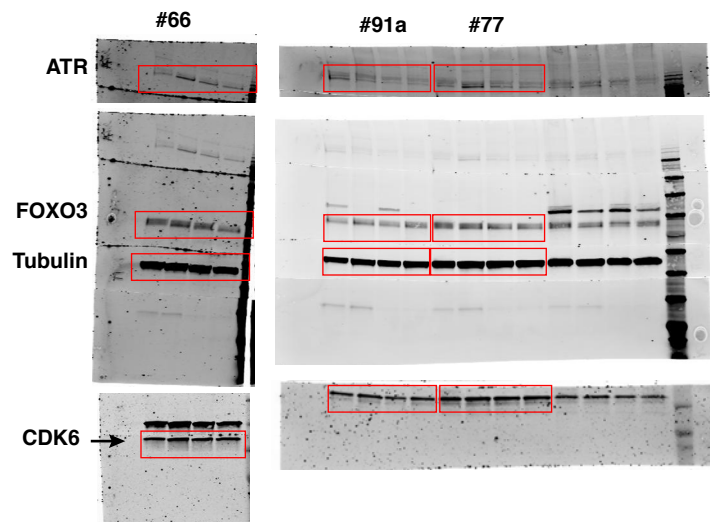

Supplement: Supplementary file 16 — Source Data for Figure 7 [file EMMM-9-1415-s014.pdf]
